# Chiral edge waves in a dance-based human topological insulator

**DOI:** 10.1126/sciadv.adh7810

**Published:** 2024-08-28

**Authors:** Matthew Du, Juan B. Pérez-Sánchez, Jorge A. Campos-Gonzalez-Angulo, Arghadip Koner, Federico Mellini, Sindhana Pannir-Sivajothi, Yong Rui Poh, Kai Schwennicke, Kunyang Sun, Stephan van den Wildenberg, Dylan Karzen, Alec Barron, Joel Yuen-Zhou

**Affiliations:** ^1^ Department of Chemistry and Biochemistry, University of California San Diego, La Jolla, CA 92093, USA.; ^2^ Orange Glen High School, Escondido, CA 92027, USA.; ^3^ Center For Research On Educational Equity, Assessment and Teaching Excellence, University of California San Diego, La Jolla, CA 92093, USA.

## Abstract

Topological insulators are insulators in the bulk but feature chiral energy propagation along the boundary. This property is topological in nature and therefore robust to disorder. Originally discovered in electronic materials, topologically protected boundary transport has since been observed in many other physical systems. Thus, it is natural to ask whether this phenomenon finds relevance in a broader context. We choreograph a dance in which a group of humans, arranged on a square grid, behave as a topological insulator. The dance features unidirectional flow of movement through dancers on the lattice edge. This effect persists when people are removed from the dance floor. Our work extends the applicability of wave physics to dance.

## Teaser

Choreographed from physics equations, a dance features people collectively behaving as a topological insulator.

## INTRODUCTION

A topological property of an object is one that is unchanged as the object undergoes continuous deformation, which includes translation, rotation, stretching/compression, and bending but excludes puncturing, tearing, and gluing (together different parts of it). More than just a theoretical concept, this notion can have real-life applications. Consider a physical material with a topological property. The latter is resistant to material imperfections that constitute continuous deformations (although not necessarily in real space). Because of this robustness, such materials, which are known as topological materials, have garnered widespread attention over the past several decades (*1*–*3*).

To date, the most studied topological material has been the topological insulator (*1*). A topological insulator is insulating in the bulk but conducting on the boundary. The earliest known topological insulators are two-dimensional electronic materials that exhibit the integer quantum Hall effect (*4*), in which the (transverse Hall) conductance along the sample edge is proportional to a nonzero integer ν, known as the Chern number (*1*, *5*). Reflecting the net number of edge states that support clockwise (or counterclockwise) current, ν and thus the edge conductance are topological properties (*5*). These characteristics of the edge are intimately related to properties of the material bulk. Such bulk-boundary correspondence is a hallmark of topological insulators.

Since their discovery, topological insulators have been observed in a plethora of other physical media (*6*). Examples include traditional wave media, both natural [e.g., oceanic and atmospheric fluids (*7*)] and synthetic [e.g., photonic (*8*, *9*) and acoustic (*10*, *11*) lattices]. Topological insulators have also been reported in settings that have less in common with electronic materials: systems governed by Newton’s equations of motion (*11*–*14*), molecular enantiomers (*15*, *16*), amorphous materials (*17*), active matter (*18*–*25*), and stochastic processes (*18*, *26*–*32*).

The ubiquity of topological insulators prompts the question of whether their physics can manifest in contexts that transcend the usual boundaries of science. In this work, we present a human topological insulator in the form of a group dance. Functioning literally as a numerical integrator of the time-dependent Schrödinger equation (TDSE), the dance features chiral motion through people along the edge of the dance floor, even when “defects” are introduced by removing dancers. In essence, this dance is distinct from those that serve as natural examples or purely qualitative representations of concepts in science and math [including seismic waves (*33*), electrical circuits (*34*), flocking (*35*), and topology (*36*)]. Thus, the dance described in this article serves both as a rigorous realization of topological edge modes and an ideal outreach activity to introduce broader audiences to the universal concepts of topological protection.

## RESULTS

**Fig. 1.**
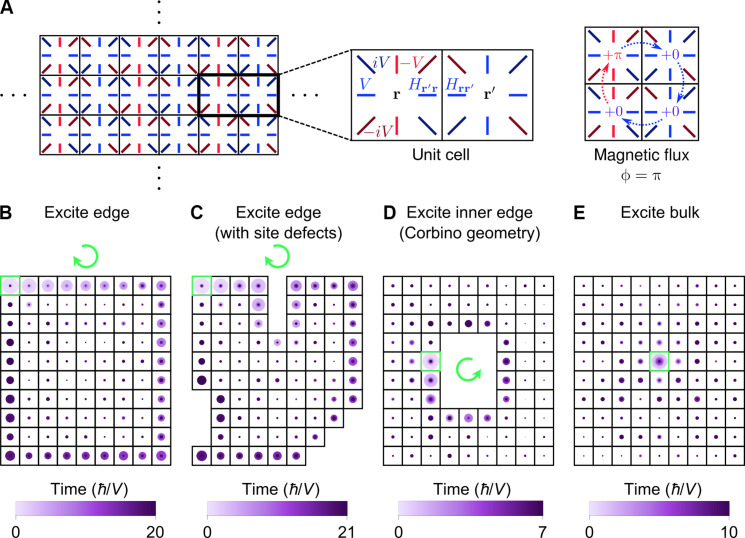
Dynamics of a model topological insulator. (**A**) Pictorial representation of the Harper-Hofstadter Hamiltonian (*H*) with NNN hopping and magnetic flux ϕ = π (Eq. 1). (**B** to **E**) Dynamics of *H* on a 10 × 9 lattice. The system is excited at a site (green box) located (B) on the edge, (C) on the edge of the lattice with site defects, (D) on the inner edge of the lattice with a 4 × 3 hole in the middle (i.e., Corbino geometry), and (E) in the bulk. Site probabilities at different times are overlaid in chronological order (i.e., later times on top). The probability of the system being at each site is represented by a circle (area ∝ probability). Excitations move unidirectionally along each edge, where the chirality of motion is indicated by a green arrow.

To begin the choreography, we consider the Harper-Hofstadter Hamiltonian (*37*, *38*) with next-nearest neighbor (NNN) coupling (*39*) and magnetic flux ϕ = π per plaquette (Fig. 1A),

$$\begin{array}{cc} H= & V\sum_{m,n}‍[(\left|m+1,n\rangle \langle m,n|+e^{i\phi m}|m,n+1\rangle \langle m,n\right|\\ & +e^{i\phi \left(m+1/2\right)}|m+1,n+1\rangle \langle m,n|\\ & +e^{i\phi \left(m-1/2\right)}|m-1,n+1\rangle \langle m,n|)+H.c.] \end{array}$$ (1)

which models an electron hopping on a square lattice in a magnetic field. The lattice sites are labeled by **r** = (*m*, *n*), and *V*(>0 here) is the magnitude of intersite coupling. Hops to a nearest neighbor (NN) occur with an amplitude of ±*V*, while hops to a NNN occur with an amplitude of ±*iV*. Here, H.c. stands for Hermitian conjugate.

The Hamiltonian *H* gives rise to several dynamical features that are characteristic of topological insulators, as shown by simulations on a finite lattice (see Materials and Methods). When exciting an edge site of a square-shaped lattice, the excitation propagates clockwise along the edge (Fig. 1B and movie S1). This chiral transport persists after introducing lattice defects of various shapes (Fig. 1C and movie S2). For a lattice with a hole in the middle, which is known as the Corbino geometry (*40*, *41*), an excitation at the inner edge moves along this edge with opposite handedness, i.e., counterclockwise (Fig. 1D and movie S3). The unidirectional conduction on the edges is drastically different from the dynamics in the bulk, in which a localized excitation diffuses with little directional selectivity (Fig. 1E and movie S4).

**Fig. 2.**
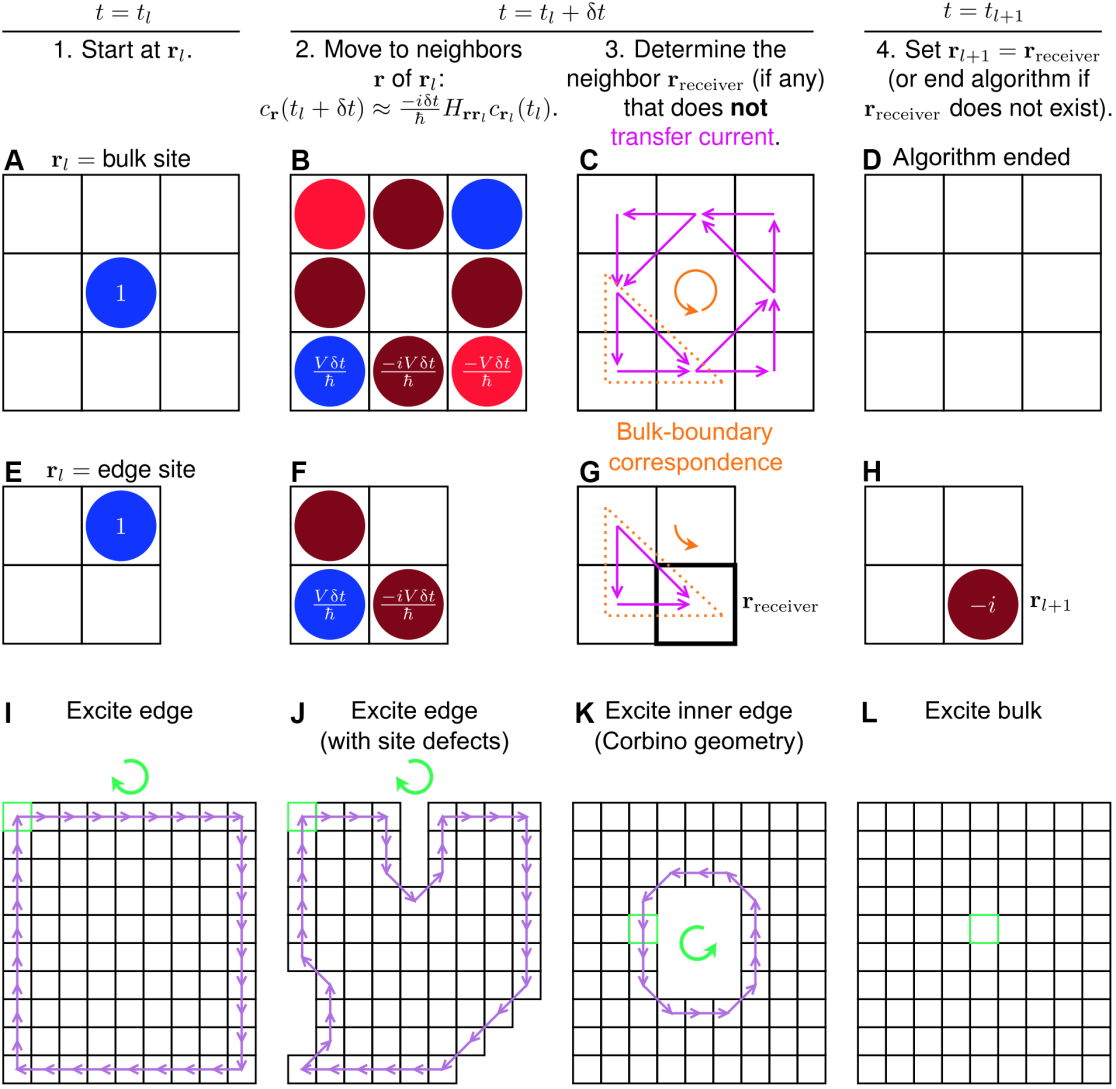
Algorithm to generate discrete-time dynamics of a topological insulator. (**A** to **H**) Illustration of the algorithm, referred to as numerical TDSE throughout the text. The wave function starts an iteration either (A to D) in the bulk or (E to H) on the edge. (A, B, E, F, and H) The probability amplitudes *c*_**r**_ are illustrated, following the same color scheme as the hopping amplitudes *H*_**rr**′_ in Fig. 1A. (C and G) For each pair of sites **r** and **r**′ such that **r** transfers current to **r**′, the current vector 〈*J*_**r**→**r**′_(*t_l_* + δ*t*)〉(**r**′ − **r**) is represented by a purple arrow. There is a bulk-boundary correspondence with respect to the current field (orange triangles) and its chirality (orange arrows). (**I** to **L**) Dynamics simulated by the algorithm, where the excitation conditions and lattice geometries are those of Fig. 1 (B to E), respectively. For each simulation, the wave function starts at the site indicated by the green box. A purple arrow represents the movement of the wave function from **r***_l_* (tail) at time step *l* to **r**_*l*+1_ (head) at time step *l* + 1. The discrete-time dynamics shows unidirectional motion along each edge, where the chirality of motion is indicated by a green arrow.

To capture such dynamics in a dance, we first present an algorithm to (approximately) propagate the wave function ∣ψ(*t*)〉 = ∑_**r**_ *c*_**r**_(*t*)∣**r**〉 in discrete time. The algorithm, hereafter referred to as “numerical TDSE,” goes as follows (Fig. 2, A to H):

1) At the *l*th time step, *t* = *t_l_*, the wave function is at site **r**_*l*_:

$$|\psi \left(t_{l}\right)\rangle =c_{r_{l}}\left(t_{l}\right)|r_{l}\rangle$$ (2)

where

$$c_{r}\left(t_{l}\right)=\left\{\begin{array}{cc} \pm 1, & \sigma \left(r_{l}\right)\;\text{even}\\ \pm i, & \sigma \left(r_{l}\right)\;\text{odd} \end{array}\right.$$ (3)

and σ(**r**) = *m* + *n* (Fig. 2, A and E).

2) Evolve the wave function forward by time δ*t* < *t*_*l*+1_ − *t_l_* and approximate the resulting state up to *O*(δ*t*):

$$|\psi (t_{l}+\delta t)\rangle \approx \left(1-\frac{i\delta t}{\hbar }H\right)|\psi (t_{l})\rangle =c_{r_{l}}(t_{l})\left(|r_{l}\rangle -\frac{i\delta t}{\hbar }\sum_{r\in N\left(r_{l}\right)}‍H_{rr_{l}}|r\rangle \right)$$ (4)

where 𝒩(**r***_l_*) is the set of neighbors (NN and NNN) of **r***_l_* (Fig. 2, B and F).

3) Determine the neighbor **r**_receiver_ (if any) of **r***_l_* that does not transfer current to any site (Fig. 2, C and G). Here, the (probability) current from **r** to **r**′ is represented by the operator $J_{r\to r\prime }=\frac{i}{\hbar }\left(H_{\mathrm{rr}\prime }|r\rangle \langle r\prime |-H_{r\prime r}|r\prime \rangle \langle r|\right)$ (*42*, *43*). We say that **r** transfers current to **r**′ if 〈*J*_**r**→**r**′_(*t_l_* + δt)〉 > 0.

4) If there is a neighbor **r**_receiver_ of **r***_l_*, reset the wave function as

$$|\psi \left(t_{l+1}\right)\rangle =\text{sgn}\left[c_{r_{\text{receiver}}}\left(t_{l}+\delta t\right)\right]|r_{\text{receiver}}\rangle$$ (5)

where sgn *z* = *z*/∣*z*∣ is the complex sign function, and **r**_*l*+1_ = **r**_receiver_ (Fig. 2H); return to step 2. If not, then the algorithm terminates (Fig. 2D).

Crucial to the numerical TDSE is its unconventional use of the current operator, enabling the probability amplitudes to interfere in step 3 and ultimately localize at **r**_receiver_ in step 4. This makes intuitive sense, since **r**_receiver_ is the “attractor/sink” of the current field at time *t_l_* + δ*t* (step 3, Fig. 2G). We have assumed that there is at most one neighbor **r**_receiver_ of **r***_l_*, which is true for the lattice geometries appearing in this work (section S2). Notice that conservation of probability, and thus current, is temporarily violated in step 2 but eventually enforced in step 4. As shown below, the algorithm can continue indefinitely for suitable initial conditions. Otherwise, the algorithm terminates, after which the wave function no longer moves, although it remains in the excited state; a similar immobilization would be achieved, for example, if we replace termination with a reset back to step 1, but this alternative would eventually result in an endless loop of the last iteration. It turns out that the algorithm is a non-Hermitian approximation of the actual quantum dynamics (section S3.2). Regardless, the topological features are preserved (*44*, *45*), as we will see below. In particular, because the current operator depends on *H* (Eq. 1) by definition (see step 3), the interference in step 3 respects the topological properties of the Hamiltonian.

In Fig. 2 (I to L), we show the dynamics generated by the numerical TDSE. The results are in excellent qualitative agreement with the exact dynamics (Fig. 1, B to E, respectively; see also movies S1 to S4, respectively). Notably, the algorithm reproduces the confinement of an edge excitation to the edge, the chirality with which this excitation moves, and the robustness of these properties to site defects. Also captured is the diagonal movement of an edge excitation as it travels around the defects (cf. Figs. 1C and 2J) and, in the Corbino geometry, past the corners of the inner edge (cf. Figs. 1D and 2K). That a bulk excitation does not move in the approximate dynamics (Fig. 2L) reflects its diffusive (and not ballistic) nature in the exact quantum dynamics (Fig. 1E). In contrast, an excitation initialized on the edge propagates indefinitely (Fig. 2, I to K). We stress that, while the edge and bulk motions produced by the numerical TDSE are very different, they arise from the same steps. In contrast, the dynamics will be the same (i.e., no propagation) starting in any bulk site and for any system size. Therefore, the algorithm allows one to qualitatively reproduce the edge and bulk dynamics of large systems using small lattices (see dance below).

Underlying the accuracy of the numerical TDSE is its use of the site currents (step 3), which give rise to the dynamical signatures of topological insulators, particularly the chiral edge motion. For an iteration starting at a bulk site (Fig. 2A), current flows to and from all neighbors of the initial site (Fig. 2C). As a result, the algorithm ends (Fig. 2, D and L), reflecting the absence of unidirectional propagation in the bulk. The current vectors form a vortex with a well-defined chirality (i.e., counterclockwise; see Fig. 2C, purple arrows). By considering an appropriate subset of this current field (Fig. 2C, orange dotted triangle), one can obtain the current field for an iteration beginning at an edge (Fig. 2G, orange dotted triangle). This subfield (Fig. 2G, purple arrows) determines the site that the wave function will occupy at the start of the next iteration (Fig. 2H). Thus, the currents of a bulk-localized wave function indicate how an edge-localized wave function would propagate. In particular, the subset relation between edge and bulk current fields (Fig. 2, G and C, orange dotted triangles) and the structure of the latter (Fig. 2C, purple arrows) explain why edge excitations are confined to the edge. Furthermore, the chirality of the bulk currents (Fig. 2C, orange arrow) is directly correlated with the chirality of the edge dynamics (Fig. 2G, orange arrow). These properties constitute a dynamical form of bulk-boundary correspondence. In particular, they closely resemble the classical picture of an electron in a magnetic field, where the circular orbits of a free particle manifest as unidirectional skipping motion along an edge (*1*).

As further evidence that the topological physics is preserved in the approximate dynamics, it can be shown that the Chern number (*1*, *5*), ν = 1 for Hamiltonian 1, naturally arises from the bulk currents. Specifically, the sum of the currents, over loops (fig. S7) enclosing an integer number of unit cells (Fig. 1A), is proportional to ν (section S4). This result, plus the correspondence between bulk currents and edge dynamics, imply that the chirality of edge propagation is exactly given by ν. Given that the Chern number is a bulk quantity, the connection between the topological invariant and bulk currents is not totally unexpected, although we are unable to provide further intuition for it at present (section S7.3).

To convert the algorithm to a dance, we have remarkably found a dynamics-preserving transformation of the wave function from the complex plane to the real numbers. To see that this mapping is possible, notice that, at all times, *t* explicitly considered in the algorithm (i.e., *t_l_*, *t_l_* + δ*t*), the probability amplitude at each **r** satisfies

$$c_{r}\in \left\{\begin{array}{cc} \mathbb{R}, & \sigma \left(r\right)\;\text{even}\\ i\mathbb{R}, & \sigma \left(r\right)\;\text{odd} \end{array}\right.$$ (6)

This property results from the choice of initial state (Eq. 3), which depends on whether σ(**r**) is even or odd; the update rule of step 4 (Eq. 4); and the structure of the Hamiltonian (Eq. 1), which has purely real NN couplings and purely imaginary NNN couplings. Moreover, since all hopping amplitudes have the same magnitude (Eq. 1), then ∣*c*_**r**_(*t_l_* + δ*t*)∣ = ∣*c*_**r**′_(*t_l_* + δ*t*)∣ for all neighboring sites **r**, **r**′ of **r***_l_*. It follows that only the signs of these coefficients are necessary to capture the dynamics (Fig. 2, I to L), where the signs are given by

$$f\left(z\right)=\left\{\begin{array}{cc} \text{sgn}\left(z\right), & z\in \mathbb{R}\\ \text{sgn}\left(z/i\right), & z\in i\mathbb{R} \end{array}\right.$$ (7)

Thus, applying *f* to all probability amplitudes *c*_**r**_ recasts the numerical TDSE in terms of real numbers (section S5), i.e., the transformed amplitudes c_**r**_′ ≡ *f*(*c*_**r**_) and “effective Hamiltonian”

$$H_{r\prime r}=\left\{\begin{array}{cc} -f\left(H_{r\prime r}\right), & \sigma \left(r\prime \right)\;\text{odd and }\sigma \left(r\right)\;\text{even}\\ f\left(H_{r\prime r}\right), & \text{else} \end{array}\right.$$ (8)

By definition, *c*_**r**_′ and $H_{r\prime r}$ each takes the values 0 and ±1. We call the reformulated algorithm “real-valued TDSE.” We emphasize that the site probabilities at times *t_l_*, and hence the dynamics (Fig. 2, I to L), remain unchanged from the numerical TDSE (section S5.3). The connections to the Chern number also survive the reformulation (section S5.4).

We proceed to choreograph a dance, which is an implementation of the real-valued TDSE. The probability amplitudes are represented by dance moves:

$$c_{r}\prime =\left\{\begin{array}{cc} 1 & \to \text{up}\\ 0 & \to \text{stand still}\\ -1 & \to \text{down} \end{array}\right.$$ (9)

**Fig. 3.**
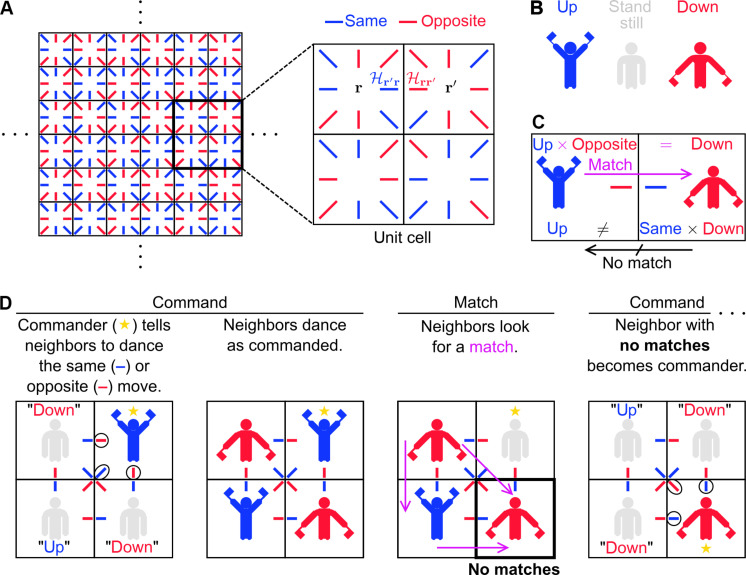
Mechanics of the dance. (**A**) Dance floor. As shown in the zoom-in of the unit cell, the squares represent the lattice sites, and the colored lines represent the matrix elements of H (Eq. 8), the “effective Hamiltonian” that generates the dance dynamics. (**B**) Dance moves. (**C**) Example of what a “match” is and is not. (**D**) Illustration of the dance steps for one round of the dance.

“Up” and “down” refer to the waving of flags with arms pointed in the indicated direction (Fig. 3B, blue and red, respectively). In contrast, “stand still” is exactly as the name suggests, with arms relaxed at the sides of the dancer (Fig. 3B, gray). The (nonzero) hopping amplitudes are represented as

$$H_{r\prime r}=\left\{\begin{array}{cc} 1 & \to \text{same}\\ -1 & \to \text{opposite} \end{array}\right.$$ (10)

The above redefinitions result in the following rules for multiplying the probability amplitudes with the hopping amplitudes

$$c_{r^{\prime }}\times H_{r\prime r}=\left\{\begin{array}{cc} \text{up}\times \text{same} & =\text{up}\\ \text{up}\times \text{opposite} & =\text{down}\\ \text{down}\times \text{same} & =\text{down}\\ \text{down}\times \text{opposite} & =\text{up} \end{array}\right.$$ (11)

With this “human representation” of the wave function and “effective Hamiltonian” H, the real-valued TDSE is readily implemented as a dance, described as follows. The dance floor (Fig. 3A) is a finite square grid, where the squares contain blue and red lines. Each square represents a lattice site **r**. The blue/red lines within that square represent the amplitudes $H_{r\prime r}$ = same/opposite of hopping to neighboring sites **r**′ (Fig. 3A, zoom-in). For each site that the electron can occupy, a dancer is placed in the corresponding square. Given this setup, the dance is performed in rounds, where each round has the steps below (Fig. 3D):

(Command) The person designated as the “commander” is dancing up or down. The commander tells each neighbor to dance in the same or opposite way, according to the line in the commander’s square that points to this neighbor (Fig. 3D, circled lines). The neighbors start dancing as commanded.

(Command-to-Match transition) The commander stands still.

(Match) Within the neighbors of the commander, each person scans across the others, looking for a “match.” As demonstrated in Fig. 3C, the person at **r** matches with the person at **r**′ if the dance move of the former, times $H_{r\prime r}$, equals the dance move of the latter, where $H_{r\prime r}$ is given by the line in square **r** that points to square **r**′.

(Match-to-Command transition) All people with a match stop dancing. If there is a person without a match, then this person continues dancing and becomes the commander; return to the Command step. If everyone has a match, the dance ends.

In a round (equivalent to an iteration of the algorithm), the site and dance move (up or down) of the commander denote the site and phase of the initially localized excitation, respectively (see steps 1 and 4 of the algorithm). The Command step (equivalent to step 2 of the algorithm) represents the spreading of the wave function to neighboring sites. The probability amplitudes at these sites interfere during the Match step (equivalent to step 3 of the algorithm) and Match-to-Command transition (equivalent to step 4 of the algorithm). Rather than having to memorize the values of $H_{r\prime r}$ for Command and Match, the dancers simply consult the blue and red lines in their respective squares.

**Fig. 4.**
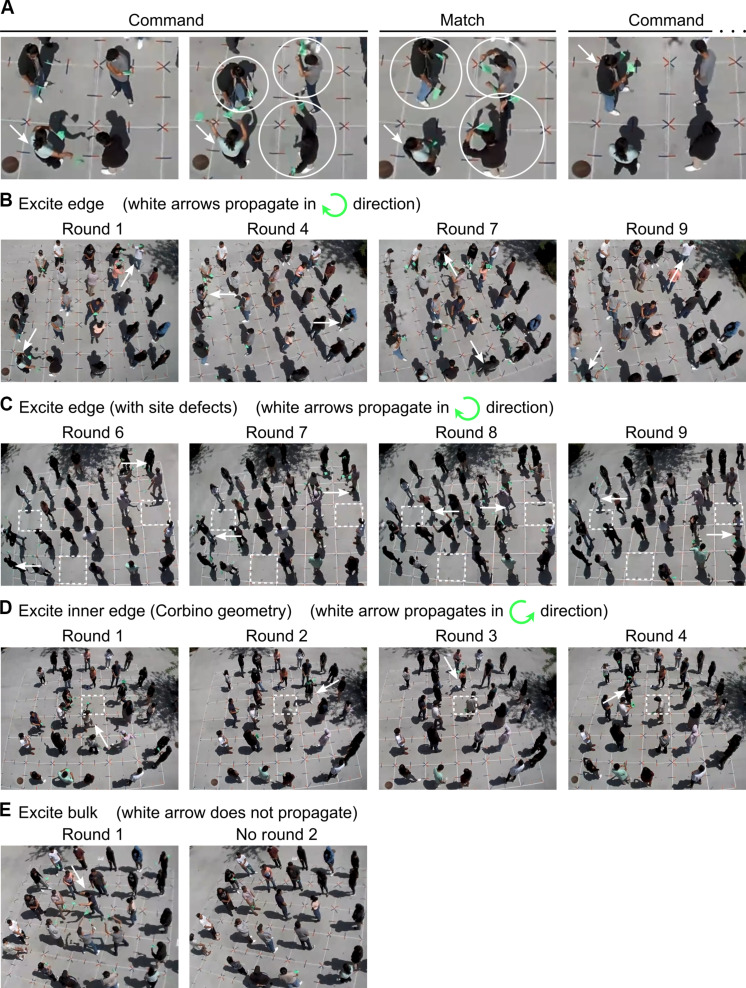
Dynamics of a dance-based human topological insulator. (**A**) Snapshots from one round of the dance. (**B** to **E**) Snapshots of the dance where the initial (round 1) dancers (commanders) are (B) on the lattice edge, (C) on the edge of a lattice with site defects, (D) on the inner edge of a lattice with a hole in the middle (i.e., Corbino geometry), and (E) in the lattice bulk. For dances where the initial dancers are on the edge, the green arrow indicates the chirality with which the dancing propagates. White dashed boxes indicate sites without a dancer. In (A) to (E), white arrows indicate commanders. Notice how the white arrows evolve according to the chirality of the corresponding green arrow. In (A), the white circles indicate neighbors (NN and NNN) of the commander who are dancing up or down.

As a science outreach event, we taught the dance to students at Orange Glen High School in Escondido, California (see Materials and Methods). Overall, the students mastered the dance steps (Fig. 4A) in under 1 hour. The students (plus some of us) then performed the dance for various initial conditions and lattice geometries (see Materials and Methods). To engage more students, some performances began with two people dancing (as commander) on the same dance floor; two concurrent dances ensued independently (see, for example, Fig. 4B and movie S5) for all but one dance round (see below). A designated leader (one of us) helped students transition between Command and Match (see Materials and Methods), similar to how the “caller” in contra dancing reminds the dancers of which figure to perform next. The performances display key dynamical features of topological insulators, namely, those generated by the algorithm on which the choreography is based. When the initial dancers are at an edge, the dancing propagates unidirectionally along this edge: clockwise on the outer edge of a square-shaped lattice (Fig. 4B and movie S5) and counterclockwise on the inner edge of a lattice with Corbino geometry (Fig. 4D and movie S7). For the former lattice, we introduced site defects by removing people at the edge of the dance floor. Still, the lattice sustains an edge-confined and clockwise-oriented “dance wave,” which maneuvers around the vacancies (Fig. 4C and movie S6) and even persists through the interference of two concurrent dances (movie S6). The dance is also robust to some forms of human error (section S6). As expected (Fig. 2L), the dance only lasts one round when an initial dancer is in the bulk (Fig. 4E and movie S8).

## DISCUSSION

In summary, we have choreographed a dance in which a group of people behave as a topological insulator. The choreography involves developing an algorithm for approximate wave function propagation and mapping the wave function first to the real numbers and then to human movements. The resulting dance, which operates as a numerical integrator of the TDSE, exhibits the salient dynamics of topological insulators. In principle, the dance dynamics can be implemented using any system that can carry out computation. This work provides a blueprint for creating a classical simulator of topological insulators. Achieving this task for additional Hamiltonians (section S7) would mark an intriguing and unique frontier at the interface of wave physics, science education, and dance. Overall, we have contributed an advancement that is both scientific and artistic, revealing a class of systems that support topological physics while inspiring content that can be expressed through dance.

## MATERIALS AND METHODS

### Calculating the dynamics generated by *H*

At time *t* = 0, the system is excited (i.e., initialized) at site **r***_I_*, and the wave function is given by

$$|\psi \left(0\right)\rangle =|r_{I}\rangle$$ (12)

To calculate the wave function at later times, we first move to the eigenbasis

$$|\psi \left(0\right)\rangle =\sum_{\alpha }‍c_{\alpha }\left(0\right)|\alpha \rangle$$ (13)

where ∣α〉 is the eigenstate of *H* with energy *E*_α_. We then calculate the wave function at time *t* as

$$|\psi \left(t\right)\rangle =\sum_{\alpha }‍c_{\alpha }\left(0\right)e^{-iE_{\alpha }t/\hbar }|\alpha \rangle$$ (14)

Changing back to the position basis,

$$|\psi \left(t\right)\rangle =\sum_{r}‍c_{r}\left(t\right)|r\rangle$$ (15)

we obtain the site probabilities |*c*_**r**_(*t*)|^2^. Fig. 1 (B to E) and movies S1 to S4 plot the site probabilities as a function of time using a time step of 0.1 *ℏ*/*V*.

### Science outreach: Topological dance

In this section, we describe the science outreach event at Orange Glen High School on 27 April 2022, in which we taught the dance to its students. A dance lesson was held during each of three physics classes in lieu of the normal class activities. In the lesson, students learned, practiced, and performed the dance. The lesson took most of the class period, which was 100 min long. Since class sizes were around 20 or less, we (the school teachers and dance instructors) joined the students in the dance performances. We recommend that the dance be carried out with at least 25 people, so that the human lattice has a well-defined bulk (i.e., people who will never dance up or down if the initial dancers are at the edge).

Before the lesson, we set up (fig. S1A) a big dance floor (6 by 6 square grid; figs. S1B and S2A) and several small dance floors (2 by 2 square grid; figs. S1C and S2C). The dance floors had grid lines made of beige masking tape (1″ thick) and squares measuring approximately 1 m by 1 m (fig. S2). Pieces of blue and red painters tape (1″ thick and 4 to 10″ long) were placed in each square according to the structure of “effective Hamiltonian” H (fig. S2; compare to Fig. 3A). In the big dance floor, the squares were enumerated from 1 to 36 (figs. S1B and S2B) to assist in the execution of the dance performances (see below).

Following a brief introduction to topological insulators and the lesson, the students were divided into groups of four. Each group moved to a practice dance floor, where one of us taught them the mechanics of the dance. The students first learned the dance moves. Figure S3 shows what the dance moves look like in real life (see cartoon version in Fig. 3B). To make the moves more distinguishable, dancers are encouraged to cover their flags with their hands when doing stand still (fig. S3B) and crouch when doing down (fig. S3D). The students then learned the Command step. After each student had a chance to practice being commander, they learned the Match step. Last, the students practiced both steps together (including the transitions between the steps) until mastery was achieved. Most students had mastered the dance steps after 30 to 45 min.

Next, the students moved to the main dance floor to rehearse and perform the dance for two to three sets of initial conditions (i.e., who the commanders are in the first round of the dance) and arrangements of students (e.g., square-shaped lattice, Corbino geometry). Accompanied by music, each performance proceeded as follows. We first called out the number(s) of the student(s) who would serve as the commanders in the first round of the dance. To begin the dance, we blew a whistle and announced “Command,” signaling for the assigned commanders to carry out Command. Once all neighbors of the commander(s) had begun dancing, we blew a whistle and announced “Match,” initiating the transition from Command to Match. After 15 to 20 s, which was enough for students to carry out Match, we blew a whistle and announced “Command” to switch to Command of the next round. This cycle was repeated for each round of the dance.

We note that the whistling simply serves to help the dancers switch between Command and Match and is not a necessary element of the dance. If there were more time allotted for the outreach event, then we could have taught the students to transition between the phases on their own.

## Supplementary Materials

### The PDF file includes:

Supplementary Text Figs. S1 to S17 Legends for movies S1 to S8 References
