# Supplementary material for: Chiral edge waves in a dance-based human topological insulator
Source: Sci Adv. 2024 Aug 28;10(35):eadh7810. doi: 10.1126/sciadv.adh7810 (PMC11352905; doi:10.1126/sciadv.adh7810)
Supplement: Supplementary file 1 — Supplementary Text Figs. S1 to S17 Legends for movies S1 to S8 References [file sciadv.adh7810_sm.pdf]

Science Advances
AAAS

Supplementary Materials for

Chiral edge waves in a dance-based human topological insulator

Matthew Du *et al.*

Corresponding author: Joel Yuen-Zhou, joelyuen@ucsd.edu

Sci. Adv. **10**, eadh7810 (2024)
DOI: 10.1126/sciadv.adh7810

The PDF file includes:

Supplementary Text
Figs. S1 to S17
Legends for movies S1 to S8
References

Other Supplementary Material for this manuscript includes the following:

Movies S1 to S8

Supplementary Text

S1 A useful property of the current operator

Consider the operator $J_{\mathbf{r}\to\mathbf{r}'}$ (see main text for definition) representing the current from $\mathbf{r}$ to $\mathbf{r}'$. With respect to wavefunction $|\psi(t)\rangle = \sum_{\mathbf{r}} c_{\mathbf{r}}(t)|\mathbf{r}\rangle$, the expectation value of this current is

$$\langle J_{\mathbf{r}\to\mathbf{r}'}(t)\rangle = \frac{2}{\hbar}\text{Im}\left[c^*_{\mathbf{r}'}(t)H_{\mathbf{r}'\mathbf{r}}c_{\mathbf{r}}(t)\right]. \tag{S1}$$

We use this property later in the supplementary text.

S2 Cases when there are multiple sites $\mathbf{r}_{\text{receiver}}$

In the numerical TDSE (see main text), we assume that there is at most one neighbor (NN or NNN) $\mathbf{r}_{\text{receiver}}$ of $\mathbf{r}_l$ that does not transfer current to another neighbor of $\mathbf{r}_l$. The assumption is true for the lattice geometries (e.g., square shaped, Corbino) employed in our simulations and dances. In this section, we discuss cases featuring multiple neighbors $\mathbf{r}_{\text{receiver}}$.

For a given $\mathbf{r}_l$, there are multiple sites $\mathbf{r}_{\text{receiver}}$ when the neighbors of $\mathbf{r}_l$ are a union of *non-neighboring* sets of sites (Fig. S4). Here, we denote sets $S_1, S_2, \ldots$ of sites as *non-neighboring* if none of $\mathbf{r}^{(1)}, \mathbf{r}^{(2)}, \ldots$ are neighbors for any $\mathbf{r}^{(1)} \in S_1, \mathbf{r}^{(2)} \in S_2, \ldots$. For a given lattice geometry, the presence of multiple sites $\mathbf{r}_{\text{receiver}}$ can occur if a site and its neighbors form an arrangement of Fig. S4.

To accommodate the case of multiple sites $\mathbf{r}_{\text{receiver}}$ in the numerical TDSE, one would need to change how the wavefunction localizes in Step 4 (see main text). One possible extension is to have the wavefunction be the normalized superposition (compare to Eq. 5)

$$|\psi(t_{l+1})\rangle \propto \sum_{\mathbf{r}_{\text{receiver}}} \text{sgn}\left[c_{\mathbf{r}_{\text{receiver}}}(t_l + \delta t)\right]|\mathbf{r}_{\text{receiver}}\rangle. \tag{S2}$$

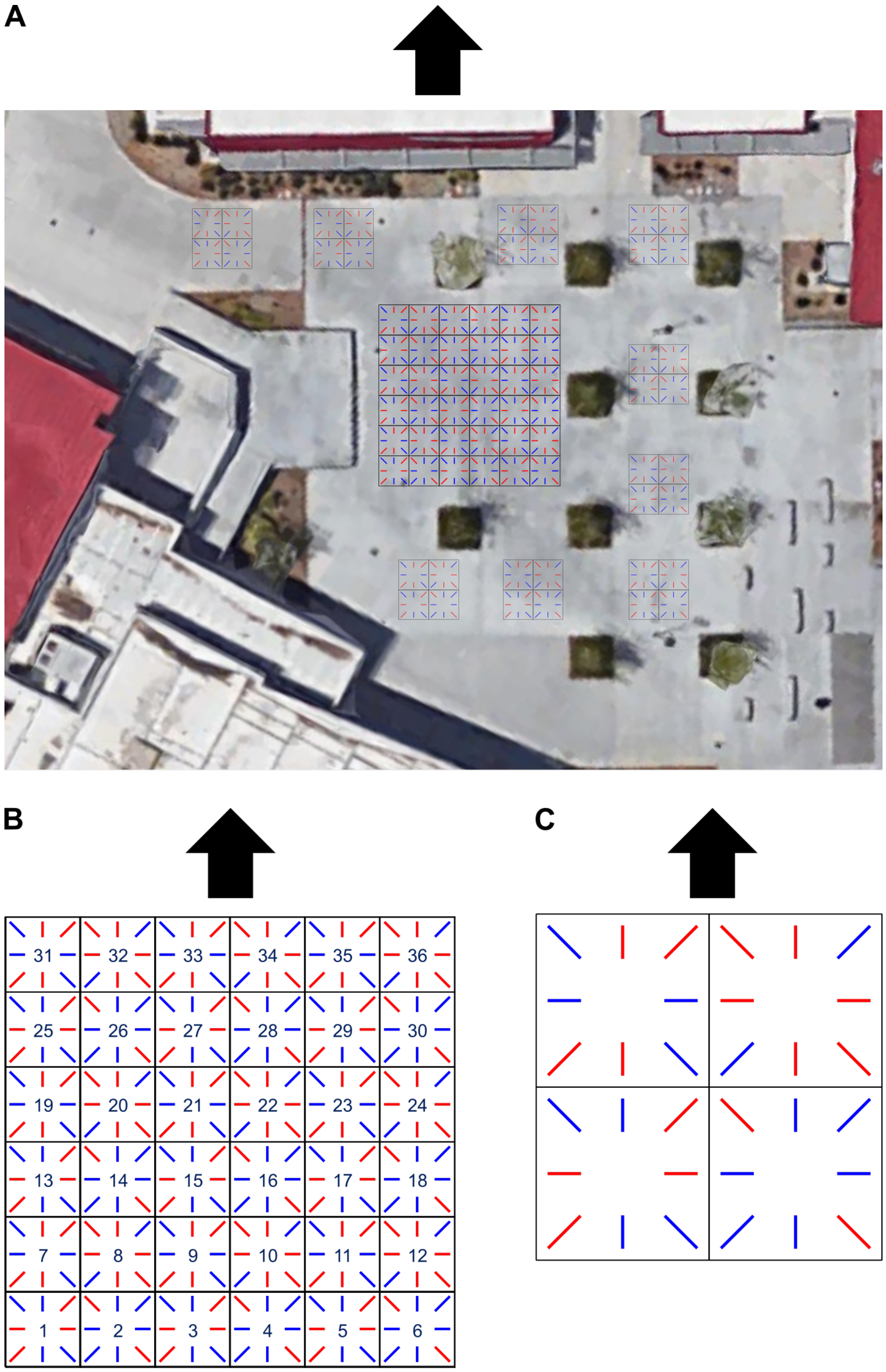

Fig. S1. Sketch of the dance floors. (**A** to **C**) Sketch of (A) the full setup of 1 big dance floor and 9 small dance floors, (B) the big dance floor, (C) a small dance floor. The arrows indicate the orientation of the dance floors in the full setup (A). Note that the lines are not drawn to scale; see Materials and Methods for the actual dimensions and Fig. S2 for the actual dance floors.

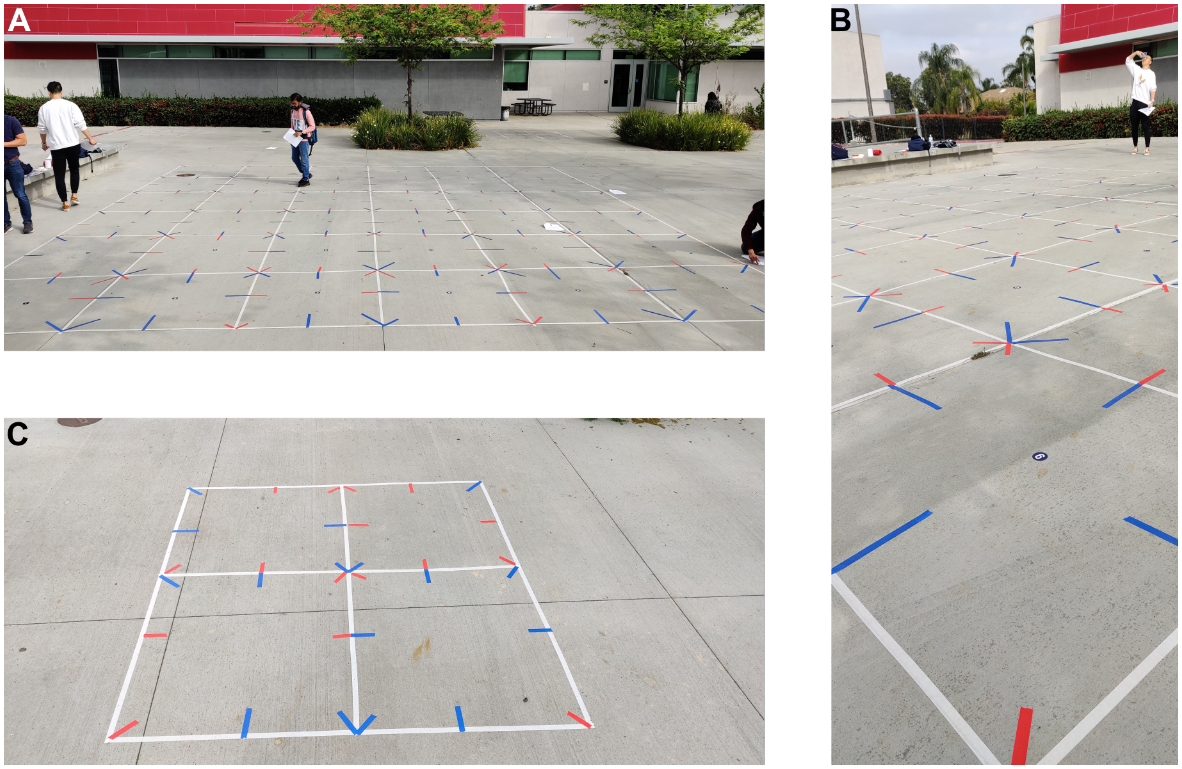

Fig. S2. Pictures of the dance floors. (**A** and **B**) Picture (A) and zoom-in (B) of the big dance floor. (**C**) Picture of a small dance floor.

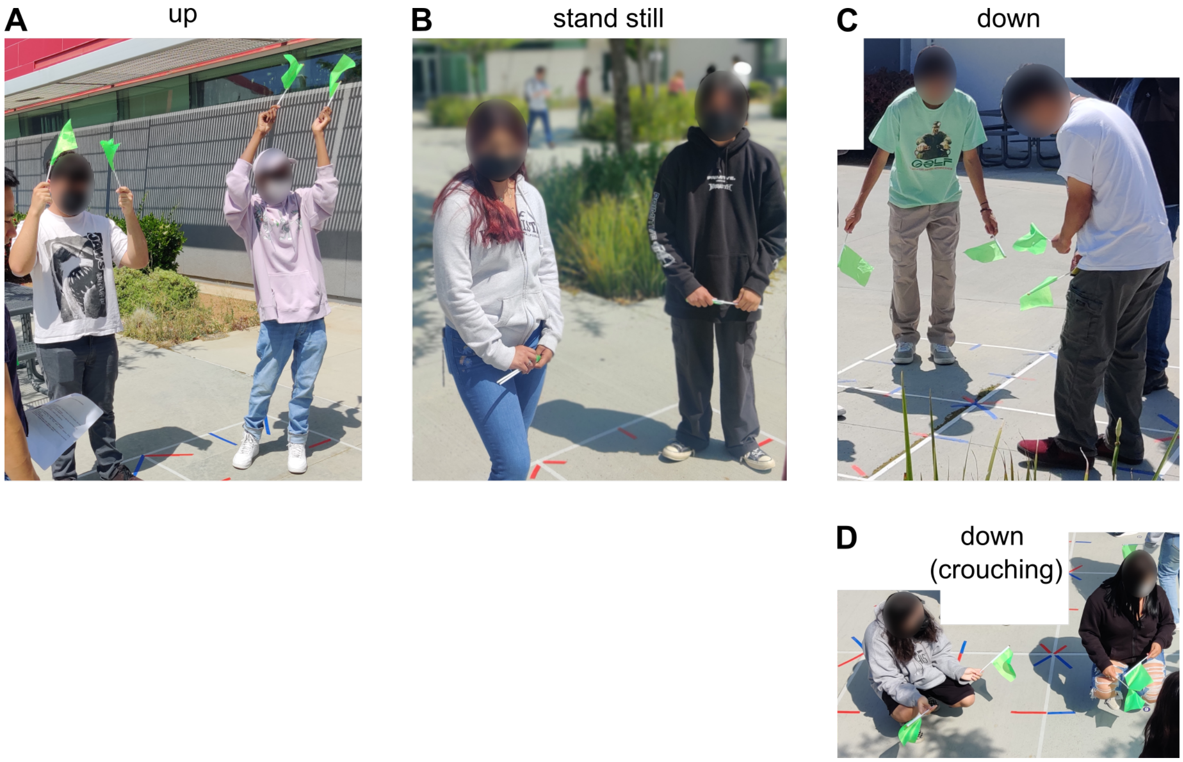

Fig. S3. Pictures of the dance moves. (**A**) Up. (**B**) Stand still. (**C**) Down (regular version, i.e., while standing). (**D**) Down while crouching.

The other steps would then be carried out in their original form. However, it is not clear how well the approximate dynamics would capture the exact dynamics.

Complications may also arise when mapping the wavefunction to real numbers, for the purpose of designing a dance. For example, a site that neighbors multiple excited sites (i.e., which have nonzero probability amplitude) can have a larger or smaller probability amplitude than a site that neighbors a single excited site. Thus, the relative magnitudes (and not just the signs) of the amplitudes may matter. This would require a modification to the function f (Eq. 7), which maps the complex amplitudes to real ones.

We defer to future studies a more detailed exploration of having multiple sites $\mathbf{r}_{\text{receiver}}$.

S3 Nonunitary time evolution operator and non-Hermitian Hamiltonian for numerical TDSE

In this section, we write down a time evolution operator corresponding to the numerical TDSE. We show that the corresponding dynamics is nonunitary. We then identify a corresponding non-Hermitian, time-periodic Hamiltonian that generates the dynamics. Thus, we should be able to study the system by applying Floquet theory, a calculation that we leave to future work.

S3.1 Nonunitary time evolution operator

Define t_0 as the initial time, $t_l = l\Delta t$ as the time at the lth time step (iteration of the algorithm), and Δt as the time step size. Introduce the time evolution operator $U(t, t_0)$, which determines the dynamics according to

$$|\psi(t)\rangle = U(t, t_0)|\psi(t_0)\rangle. \tag{S3}$$

For the numerical TDSE, we can write

$$U(t, t_0) = \mathcal{U}\left((t - t_0) \mod \Delta t\right) \left[\mathcal{U}(\Delta t)\right]^{\lfloor (t-t_0)/\Delta t \rfloor}, \tag{S4}$$

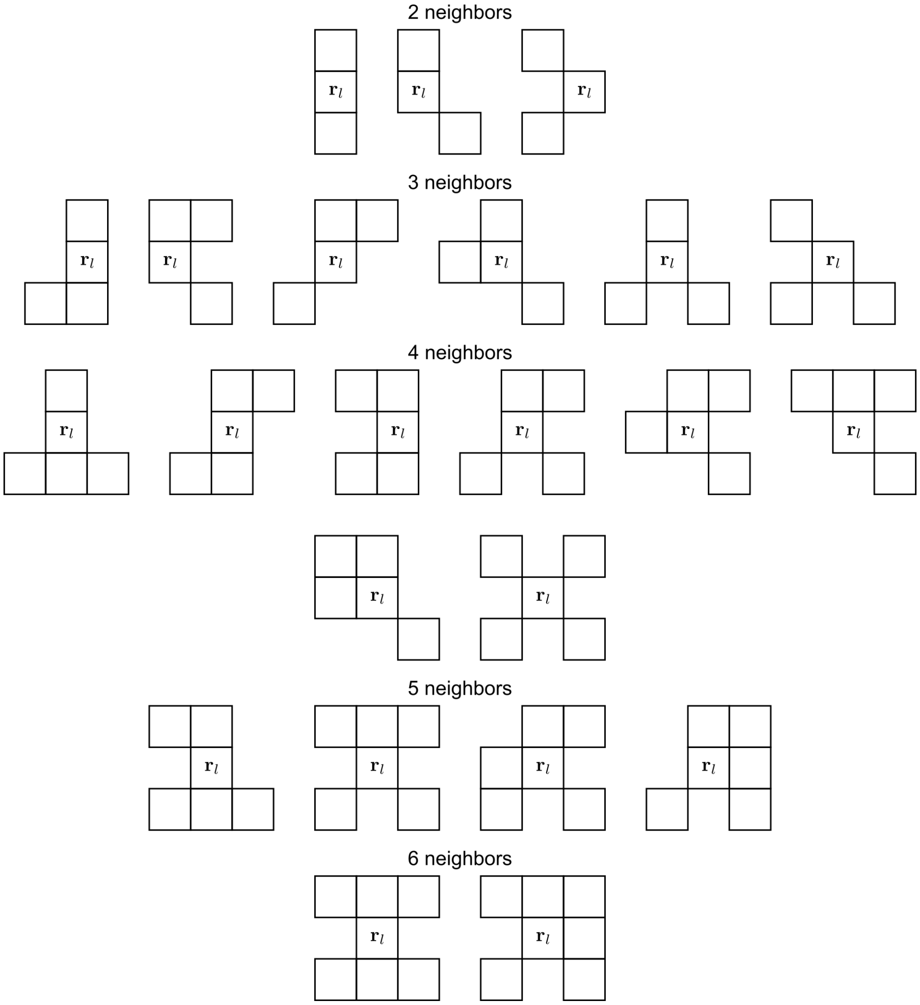

Fig. S4. Site arrangements leading to multiple sites $\mathbf{r}_{\text{receiver}}$. Arrangements of sites surrounding $\mathbf{r}_l$ where the neighbors of $\mathbf{r}_l$ are a union of non-neighboring sets of sites, leading to multiple sites $\mathbf{r}_{\text{receiver}}$. The arrangements are listed in increasing order of the number of neighbors surrounding $\mathbf{r}_l$.

where $\lfloor \cdot \rfloor$ is the floor function. The operator

$$\mathcal{U}(t) = \begin{cases} 1, & 0 \le t < \delta t, \\ \mathcal{U}_2, & \delta t \le t < \Delta t, \\ \mathcal{U}_{3-4}\mathcal{U}_2, & t = \Delta t, \end{cases} \tag{S5}$$

determines the evolution within a single time step, $t \in [0, \Delta t]$. Step 2 of the algorithm is carried out by

$$\mathcal{U}_2 = 1 - \frac{i\delta t}{\hbar} H. \tag{S6}$$

Steps 3-4 are carried out by the operator $\mathcal{U}_{3-4}$, which acts on a state $|\phi\rangle$ according to

$$\mathcal{U}_{3-4}|\phi\rangle = \mathrm{sgn}\left(c_{\mathbf{r}_{\mathrm{receiver}}}\right)|\mathbf{r}_{\mathrm{receiver}}\rangle, \tag{S7}$$

where $\mathbf{r}_{\mathrm{receiver}}$ and $c_{\mathbf{r}_{\mathrm{receiver}}}$are determined by applying these two steps to $|\phi\rangle$. If no site is $\mathbf{r}_{\mathrm{receiver}}$, then $\mathcal{U}_{3-4} = \mathcal{U}_2^{-1}$ (note: by Eq. S6, $\mathcal{U}_2$ is invertible due to the Hermiticity of H), which reverts the state back to what it was in the most recent instance of Step 1 and (see main text) can be viewed as having largely the same effect (i.e., halting of propagation) as the termination of the algorithm. We note that the action of $\mathcal{U}_{3-4}$ is well defined only when the opeartor acts on states generated by Step 2 from a localized excitation, i.e., $|\phi\rangle \propto \mathcal{U}_2|\mathbf{r}\rangle$ for some site $\mathbf{r}$; defining how $\mathcal{U}_{3-4}$ transforms superpositions of such states could require, for example, extending Steps 3 and 4 to handle delocalized initial excitations (see Step 1 in main text) and multiple $\mathbf{r}_{\mathrm{receiver}}$ sites; hence $\mathcal{U}_{3-4}$ is not a linear operator.

It is straightforward to show that $U(t, t_0)$ (Eq. S4) produces nonunitary time evolution. Using the Hermiticity of H (Eq. 1), we find that $\mathcal{U}_2\mathcal{U}_2^\dagger = 1 + \delta t^2 H^2/\hbar^2 \neq 1$, i.e., $\mathcal{U}_2$ is nonunitary. Given the fact that there exist multiple $|\phi\rangle$ which yield the same $|\mathbf{r}_{\mathrm{receiver}}\rangle$, we conclude that $\mathcal{U}_{3-4}$ does not have a well-defined inverse, implying that $\mathcal{U}_{3-4}$ is nonunitary. From the nonunitarity of $\mathcal{U}_2$ and $\mathcal{U}_{3-4}$, as well as Eqs. S4-S5, it can be readily seen that $U(t, t_0)$ is nonunitary for general time t.

S3.2 Non-Hermitian, time-periodic Hamiltonian

By inspection of Eqs. S4-S5, we can write the time evolution operator (Eq. S4) for the numerical TDSE as a Dyson series (*46*) or a positive-time ordered exponential (*47*),

$$
\begin{aligned}
U(t,t_0) &= 1 + \sum_{k=1}^{\infty} \left(\frac{-i}{\hbar}\right)^k \int_{t_0}^{t} dt_1 \int_{t_0}^{t_1} dt_2 \cdots \int_{t_0}^{t_{k-1}} dt_k \mathsf{H}(t_1)\mathsf{H}(t_2)\cdots\mathsf{H}(t_k) \\
&\equiv \exp_+\left[-\frac{i}{\hbar}\int_{t_0}^{t} d\tau\, \mathsf{H}(\tau)\right],
\end{aligned}
$$

where

$$
\mathsf{H}(t) = i\hbar\delta\left([(t-t_0) \mod \Delta t] - \delta t\right)\ln\mathcal{U}_2 + i\hbar\delta\left((t-t_0) \mod \Delta t\right)\ln\mathcal{U}_{3-4}
$$

is the corresponding Hamiltonian. Note that $\mathsf{H}(t)$ is periodic in time with period Δt. Furthermore, by the nonunitarity of $\mathcal{U}_2$ and $\mathcal{U}_{3-4}$, we find that $\mathsf{H}(t)$ is also non-Hermitian for general t.

S4 Connection between the numerical TDSE and the Chern number

Consider the currents in Step 3 of the numerical TDSE, the algorithm presented in the main text, when a bulk site is excited (Fig. 2C). Here, we show that the Chern number of the underlying Hamiltonian emerges naturally when calculating the currents involved in the dance.

First, consider a more general form (*39*) (see text above their Eq. 5.1) of our Hamiltonian (Eq. 1):

$$
\begin{aligned}
H = \sum_{m,n} \Big[&\big(V_a|m+1,n\rangle\langle m,n| + V_b e^{i\phi m}|m,n+1\rangle\langle m,n| \\
&+ V_c e^{i\phi(m+1/2)}|m+1,n+1\rangle\langle m,n| \\
&+ V_c' e^{i\phi(m-1/2)}|m-1,n+1\rangle\langle m,n|\big) + \text{H.c.}\Big],
\end{aligned} \tag{S8}
$$

where $\phi = \pi$, $V_a > 0$, and $V_b, V_c, V_c' \in \mathbb{R}$. The (magnetic) unit cell of the system is shown in Fig. S5.

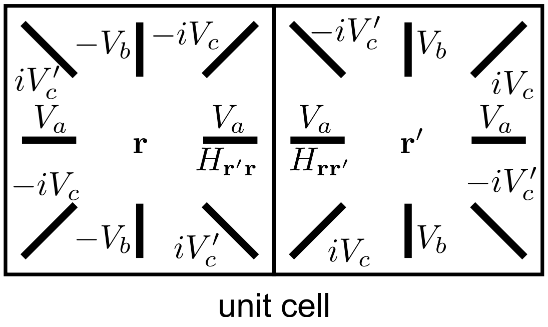

Fig. S5. Unit cell of generalized Hamiltonian. Unit cell of system described by Hamiltonian S8, consisting of two sites $\mathbf{r}$ and $\mathbf{r}'$.

Now turn to Algorithm 1. Suppose the lth iteration begins with the excitation in a bulk site $\mathbf{r}_l$ (Fig. 2A of manuscript). Without loss of generality, let $\mathbf{r}_l = (0, 0)$. In Step 3, the current from a (nearest or next-nearest) neighbor $\mathbf{r}$ of $(0, 0)$ to another neighbor $\mathbf{r}'$ can be expressed as (Eqs. 4 and S5 of manuscript)

$$\langle J_{\mathbf{r}\to\mathbf{r}'}\rangle \equiv \langle\psi(t_l + \delta t)|J_{\mathbf{r}\to\mathbf{r}'}|\psi(t_l + \delta t)\rangle = \frac{2(\delta t)^2}{\hbar^3}\mathrm{Im}\left(H_{(0,0)\mathbf{r}'}H_{\mathbf{r}'\mathbf{r}}H_{\mathbf{r}(0,0)}\right). \quad \text{(S9)}$$

Figs. S6A and S6B show the couplings and currents, respectively, for $(0, 0)$ and its neighbors.

Consider counterclockwise-oriented loops $L = \mathbf{r}_{(1)} \to \mathbf{r}_{(2)} \to \cdots \to \mathbf{r}_{(M)} \circlearrowleft$ ("$\circlearrowleft$" means go back to $\mathbf{r}_{(1)}$) passing at most once through neighbors $\mathbf{r}_{(1)}, \ldots, \mathbf{r}_{(M)}$ of $(0, 0)$, encircling $(0, 0)$, and enclosing an area equal to an integer multiple of unit cells. There are 2 such loops: (Fig. S7A)

$$L_1 = (1, 0) \to (0, 1) \to (-1, 0) \to (0, -1) \circlearrowleft, \quad \text{(S10)}$$

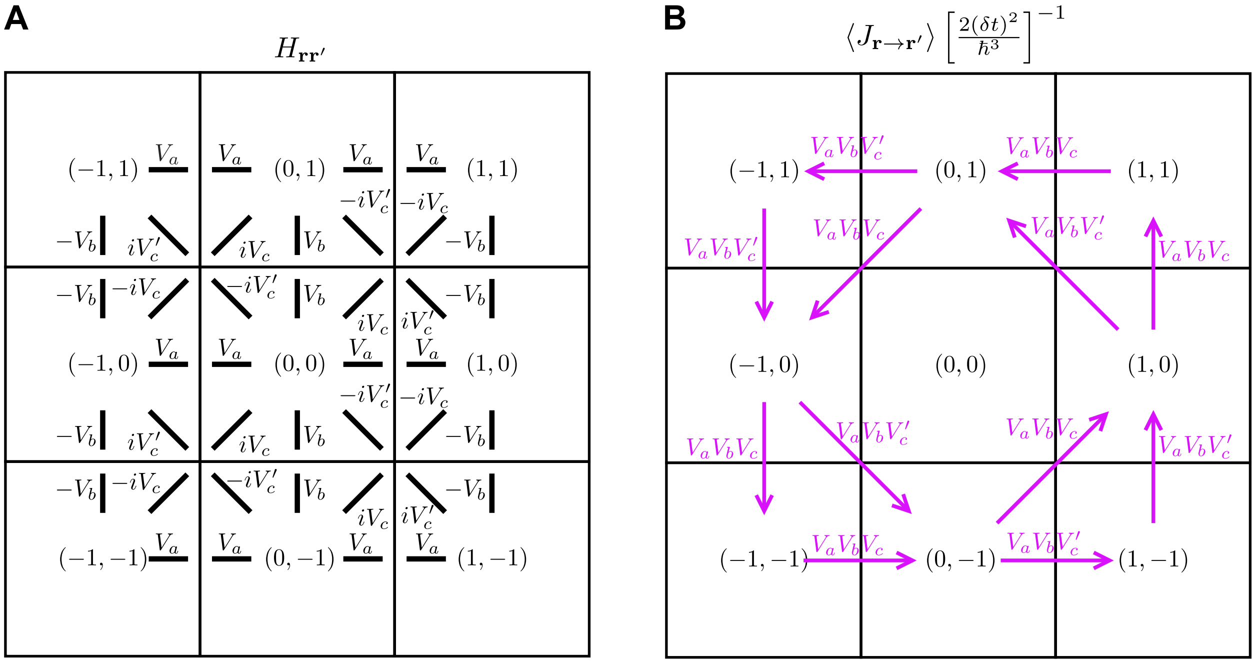

Fig. S6. Couplings and associated currents. (A) Couplings ($H_{\mathbf{rr}'}$) and (B) currents ($\langle J_{\mathbf{r}\to\mathbf{r}'}\rangle \left[\frac{2(\delta t)^2}{\hbar^3}\right]^{-1}$) for the site (0,0) and its neighbors.

a square enclosing 1 unit cell, and (Fig. S7B)

$$L_2 = (1,0) \to (1,1) \to (0,1) \to (-1,1) \to (-1,0) \to (-1,-1) \to (0,-1) \to (1,-1) \circlearrowleft, \tag{S11}$$

a square enclosing 2 unit cells.

Define

$$S_L = \sum_{i=1}^{M} \langle J_{\mathbf{r}_{(i)}\to\mathbf{r}_{(i+1)}} \rangle \tag{S12}$$

as the sum of the currents over loop L, where $\mathbf{r}_{(M+1)} = \mathbf{r}_{(1)}$. Then (Fig. S7A)

$$S_{L_1} = 2V_aV_b\left(V_c + V_c'\right) \tag{S13}$$

and (Fig. S7B)

$$S_{L_2} = 4V_aV_b\left(V_c + V_c'\right). \tag{S14}$$

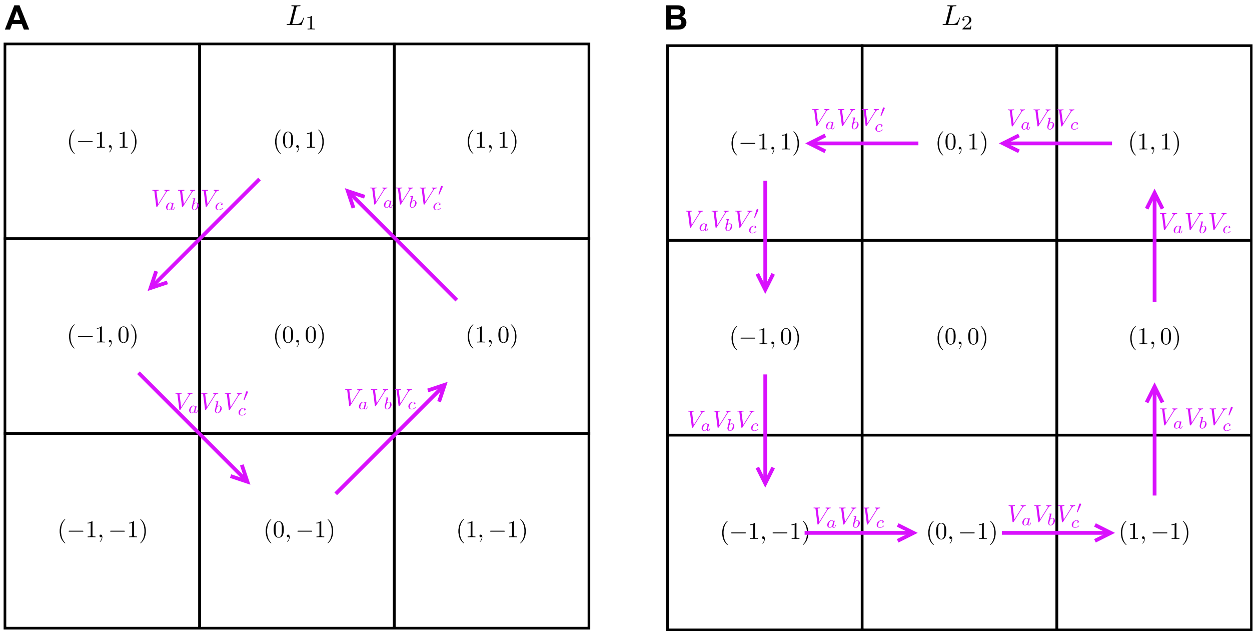

Fig. S7. Loops and associated currents. Loops (a) L_1 and (b) L_2. The arrows are labeled with their respective currents ($\langle J_{\mathbf{r}\to\mathbf{r}'}\rangle \left[\frac{2(\delta t)^2}{\hbar^3}\right]^{-1}$), as reproduced from Fig. S6B.

Since $V_a > 0$, we thus have

$$\mathrm{sgn}(S_{L_1}) = \mathrm{sgn}(S_{L_2}) = \nu, \tag{S15}$$

where (*39*) (see their Eq. 5.22)

$$\nu = \mathrm{sgn}[V_b(V_c + V_c')] \tag{S16}$$

is the Chern number, which clearly showcases the chirality (counterclockwise or clockwise) of the edge dynamics (Fig. 2, I to L), given the correspondence between the bulk currents and the edge dynamics (see main text).

S5 Real-valued TDSE: generating real-valued, discrete-time dynamics of a topological insulator

The numerical TDSE, the algorithm in the main text, propagates the (complex-valued) wavefunction in discrete time for the model topological insulator with (complex-valued) Hamilto-

nian H (Eq. 1). In this section, we present the real-valued TDSE, the algorithm that results from transforming the probability amplitudes according to $c_{\mathbf{r}} \to c'_{\mathbf{r}} = f(c_{\mathbf{r}})$, where f is the real-valued function defined in Eq. 7. So, this algorithm is written in terms of real-valued quantities. The real-valued TDSE is described in Section S5.1 and derived from the numerical TDSE in Section S5.2. We then show that the transformed algorithm has the same dynamics (Section S5.3) and connections to the Chern number (Section S5.4) as the original algorithm. These correspondences therefore exist between the numerical TDSE and the dance, which is an implementation of the real-valued TDSE.

S5.1 Algorithm

Here are the steps of the real-valued TDSE (Fig. S8):

1. At the lth time step, $t = t_l$, the wavefunction is at site $\mathbf{r}_l$:

$$|\psi(t_l)\rangle = c'_{\mathbf{r}_l}(t_l)|\mathbf{r}_l\rangle = \pm|\mathbf{r}_l\rangle. \tag{S17}$$

2. Evolve the wavefunction forward by time $\delta t < t_{l+1} - t_l$:

$$|\psi(t_l + \delta t)\rangle = c'_{\mathbf{r}_l}(t_l)\left(|\mathbf{r}_l\rangle + \sum_{\mathbf{r}\in\mathcal{N}(\mathbf{r}_l)} \mathcal{H}_{\mathbf{r}\mathbf{r}_l}|\mathbf{r}\rangle\right). \tag{S18}$$

3. Determine the neighbor $\mathbf{r}_{\text{no match}}$ (if any) of $\mathbf{r}_l$ that does not *match* with another neighbor of $\mathbf{r}_l$. We say that $\mathbf{r}$ *matches* with $\mathbf{r}'$ if the probability amplitude of the former equals that of the latter after multiplication by $\mathcal{H}_{\mathbf{r}'\mathbf{r}}$, i.e., $\mathcal{H}_{\mathbf{r}'\mathbf{r}}c'_{\mathbf{r}}(t_l + \delta t) = c'_{\mathbf{r}'}(t_l + \delta t)$.

4. If there is a neighbor $\mathbf{r}_{\text{no match}}$ of $\mathbf{r}_l$, reset the wavefunction as

$$|\psi(t_{l+1})\rangle = c'_{\mathbf{r}}(t_l + \delta t)|\mathbf{r}_{\text{no match}}\rangle \tag{S19}$$

   and $\mathbf{r}_{l+1} = \mathbf{r}_{\text{no match}}$; return to Step 2. If not, the algorithm terminates.

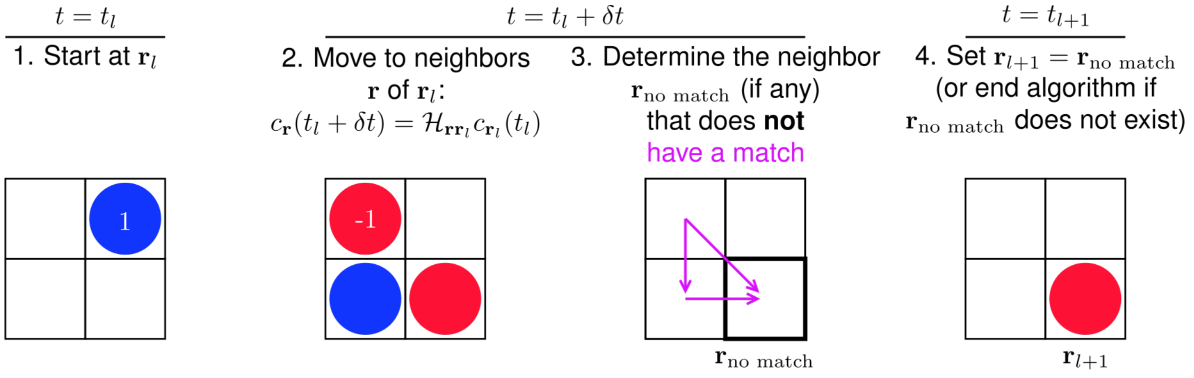

Fig. S8. Real-valued TDSE. Illustration of the algorithm referred to as "real-valued TDSE".

S5.2 Derivation

In this section, we show that applying f (Eq. 7) to the probability amplitudes $c_{\mathbf{r}}$ allows us to write each step of the numerical TDSE as the same step of the real-valued TDSE.

The derivation goes as follows:

1. By definition of $c_{\mathbf{r}_l}(t_l)$ (Eq. 3),

$$f\left(c_{\mathbf{r}_l}(t_l)\right) = \pm 1. \tag{S20}$$

   Comparing this equation to Eq. S17 for $|\psi(t_l)\rangle$ of the real-valued TDSE, we see that applying f to all $c_{\mathbf{r}}(t_l)$ converts Step 1 of the numerical TDSE to the same step of the real-valued TDSE.

2. Notice that H (Eq. 1) has purely real NN couplings and purely imaginary NNN couplings, i.e.,

$$H_{\mathbf{r}\mathbf{r}'} \in \begin{cases} \mathbb{R}, & \sigma(\mathbf{r}) \text{ even, } \sigma(\mathbf{r}') \text{ odd,} \\ \mathbb{R}, & \sigma(\mathbf{r}) \text{ odd, } \sigma(\mathbf{r}') \text{ even,} \\ i\mathbb{R}, & \sigma(\mathbf{r}) \text{ even, } \sigma(\mathbf{r}') \text{ even,} \\ i\mathbb{R}, & \sigma(\mathbf{r}) \text{ odd, } \sigma(\mathbf{r}') \text{ odd.} \end{cases} \tag{S21}$$

Using this equation, one can show that (see Eq. 4)

$$c_{\mathbf{r}}(t_l+\Delta t) = f\left(c_{\mathbf{r}_l}(t_l)\right)\frac{\delta t}{\hbar}\times\begin{cases}\mathrm{Re}H_{\mathbf{r}\mathbf{r}_l}, & \sigma(\mathbf{r})\text{ even, }\sigma(\mathbf{r}_l)\text{ odd,}\\ -i\mathrm{Re}H_{\mathbf{r}\mathbf{r}_l}, & \sigma(\mathbf{r})\text{ odd, }\sigma(\mathbf{r}_l)\text{ even,}\\ \mathrm{Im}H_{\mathbf{r}\mathbf{r}_l}, & \sigma(\mathbf{r})\text{ even, }\sigma(\mathbf{r}_l)\text{ even,}\\ i\mathrm{Im}H_{\mathbf{r}\mathbf{r}_l}, & \sigma(\mathbf{r})\text{ odd, }\sigma(\mathbf{r}_l)\text{ odd}\end{cases} \tag{S22}$$

for $\mathbf{r}\in\mathcal{N}(\mathbf{r}_l)$. It follows that

$$f\left(c_{\mathbf{r}}(t_l+\delta t)\right) = f\left(c_{\mathbf{r}_l}(t_l)\right)\mathcal{H}_{\mathbf{r}\mathbf{r}_l}, \quad \mathbf{r}\in\mathcal{N}(\mathbf{r}_l), \tag{S23}$$

where $\mathcal{H}$ is defined in Eq. 8. Moreover, since $c_{\mathbf{r}_l}(t_l+\delta t) = c_{\mathbf{r}_l}(t_l)$ (Eq. 4), then

$$f\left(c_{\mathbf{r}_l}(t_l+\delta t)\right) = f\left(c_{\mathbf{r}_l}(t_l)\right). \tag{S24}$$

Comparing Eqs. S23-S24 to Eq. S18 for $|\psi(t_l)\rangle$ of the real-valued TDSE, we see that applying f to all $c_{\mathbf{r}}(t_l+\delta t)$ converts Step 2 of the numerical TDSE to the same step of the real-valued TDSE.

3. Using Eq. S1, property S21 of H, and definition 8 of $\mathcal{H}$, we can write the current at time $t_l+\delta t$ from neighbor $\mathbf{r}$ of $\mathbf{r}_l$ to another neighbor $\mathbf{r}'$ of $\mathbf{r}_l$ as

$$\langle J_{\mathbf{r}\to\mathbf{r}'}(t+\delta t)\rangle = \frac{2(\delta t)^2}{\hbar^3} f\left(c_{\mathbf{r}'}(t+\delta t)\right)\mathcal{H}_{\mathbf{r}'\mathbf{r}} f\left(c_{\mathbf{r}}(t+\delta t)\right). \tag{S25}$$

Since $|f\left(c_{\mathbf{r}'}(t+\delta t)\right)| = |\mathcal{H}_{\mathbf{r}'\mathbf{r}}| = |f\left(c_{\mathbf{r}}(t+\delta t)\right)| = 1$, the condition $\langle J_{\mathbf{r}\to\mathbf{r}'}(t+\delta t)\rangle > 0$ is equivalent to $\frac{\hbar^3}{2(\delta t)^2}\langle J_{\mathbf{r}\to\mathbf{r}'}(t+\delta t)\rangle = 1$, or

$$\mathcal{H}_{\mathbf{r}'\mathbf{r}} f\left(c_{\mathbf{r}}(t+\delta t)\right) = f\left(c_{\mathbf{r}'}(t+\delta t)\right). \tag{S26}$$

With this result and renaming $\mathbf{r}_{\text{receiver}}$ as $\mathbf{r}_{\text{no match}}$, we can rewrite Step 3 of the numerical TDSE as the same step of the real-valued TDSE.

4. Using

$$f\left(\mathrm{sgn}\left[c_{\mathbf{r}}(t_l+\delta t)\right]\right) = f\left(c_{\mathbf{r}}(t_l+\delta t)\right) \tag{S27}$$

for all $\mathbf{r}$ and renaming $\mathbf{r}_{\text{receiver}}$ as $\mathbf{r}_{\text{no match}}$,we can rewrite Step 4 of the numerical TDSE as the same step of the real-valued TDSE.

S5.3 Proof: the real-valued TDSE has the same dynamics as the numerical TDSE

Here, we prove that the dynamics (i.e., the wavefunction at each t_l) of the real-valued TDSE is the same (up to a global phase) as the dynamics (Fig. 2, I to L) of the numerical TDSE.

Consider the lth time step (i.e., iteration) of the algorithms. Step 3 of the numerical TDSE (see main text) can be rewritten as follows: determine the neighbor $\mathbf{r}(\equiv \mathbf{r}_{\text{receiver}})$ of $\mathbf{r}_l$ such that $\langle J_{\mathbf{r}\to\mathbf{r}'}(t+\delta t)\rangle > 0$ for no site $\mathbf{r}'$, where $\langle J_{\mathbf{r}\to\mathbf{r}'}(t+\delta t)\rangle$ is computed from $|\psi(t_l+\delta t)\rangle$ (Eq. 4), the wavefunction resulting from Steps 1 and 2. Similarly, Step 3 of the real-valued TDSE (Section S5.1) can be rephrased as follows: determine the neighbor $\mathbf{r}(\equiv \mathbf{r}_{\text{match}})$ of $\mathbf{r}_l$ such that $\mathcal{H}_{\mathbf{r}\mathbf{r}'} f\left(c_{\mathbf{r}}(t_l+\delta t)\right) = f\left(c_{\mathbf{r}'}(t_l+\delta t)\right)$, where the amplitudes $c_{\mathbf{r}}$ correspond to $|\psi(t_l+\delta t)\rangle$ *of the numerical TDSE*. As noted in item 3 of Section S5.2, the criterion for determining $\mathbf{r}$ is equivalent for both algorithms. Thus, reverting to the original wording of the algorithms, either

1. $\mathbf{r}_{\text{receiver}}$ of the numerical TDSE is equal to $\mathbf{r}_{\text{match}}$ of the real-valued TDSE, or

2. neither $\mathbf{r}_{\text{receiver}}$ nor $\mathbf{r}_{\text{match}}$ exist.

In Case 1, the wavefunction at the next time step, $|\psi(t_{l+1})\rangle$, is the same (up to a global phase) for both algorithms, as seen by comparing Step 4 of each algorithm. In Case 2, the algorithm terminates for both numerical and real-valued TDSEs.

Therefore, the real-valued TDSE has the same dynamics as the numerical TDSE.

S5.4 Connections to the Chern number

In Section S4, we found that the Chern number naturally emerges from the currents computed in Step 3 of the numerical TDSE (see main text). This result, and the dynamical bulk-boundary correspondence of the numerical TDSE (see main text), imply that the chirality of the edge dynamics is given by the Chern number. However, since the real-valued TDSE (Section S5.1) is

obtained by applying a nontrivial transformation (f, Eq. 7) to the wavefunction in the numerical TDSE, one is led to ask: are the connections to the Chern number maintained in the real-valued TDSE? Below, we answer the question in the affirmative.

Consider Step 3 of both algorithms. As discussed in Section S5.3, the criterion for determining $\mathbf{r}_{\text{match}}$in the real-valued TDSE (Section S5.1) is equivalent to that for determining $\mathbf{r}_{\text{receiver}}$ in the numerical TDSE (see main text). Thus, Step 3 of the real-valued TDSE implicitly carries out the same calculation of currents as in the numerical TDSE. Because of this identification, the dynamic bulk-boundary correspondence (see main text) and the connection to the Chern number (Section S5.4) also hold for the real-valued TDSE. Hence, in this algorithm too, the chirality of the edge dynamics is given by the Chern number. Trivially, the Chern number also characterizes such edge chirality in the dance, which is an implementation of the real-valued TDSE.

S6 Robustness of the dance to human error

In this section, we briefly explore the robustness of the dance to human error. It is hard to say, in general, how such errors affect the dance. However, we can comment on specific types error. The discussion below highlights that the dance is robust to some forms of error but not all.

For example, suppose that someone incorrectly identifies a match with one neighbor (but does everything correctly thereafter). If there actually is a match with another neighbor, then the error would not affect the dance. In contrast, if there actually is no match, then the incorrect match would cause the dance to end when it really should not.

We can also consider the opposite type of error, where someone misses a match (but does everything correctly thereafter). In this case, an extra commander would arise in the next round of the dance; this additional dancer need not affect the chirality nor the edge-localized nature of the dynamics, as demonstrated in the dance performances that begin with two commanders

(see Fig. 4, B-D, and accompanying Movies S5-S6).

S7 Extending the dance to other Hamiltonians

Throughout this work, we have choreographed a dance to approximate the TDSE for the Harper-Hofstadter Hamiltonian with next-nearest-neighbor coupling (Eq. 1) (*39*). In this section, we discuss the extension to other Hamiltonians.

S7.1 Criteria for applying the approach reported here

Using the approach reported here, we should be able to create a dance for a number of Hamiltonians where

1. each neighbor of a bulk site is coupled to at least one other neighbor,
2. the phases of the couplings ($H_{\mathbf{rr}'}$) are either purely real and purely imaginary,
3. on-site energies are zero (i.e., $H_{\mathbf{rr}} = 0$),
4. for general excitations, at most one site is determined to be $\mathbf{r}_{\text{receiver}}$ in Step 3 of the numerical TDSE,
5. in the numerical TDSE, the amplitude at each site remains either real or imaginary at all times (i.e., t_l and $t_l + \delta t$).

Conditions 1 and 4 are a prerequisite for the numerical TDSE (see main text) to work. As we discuss in Section S7.4, Condition 3 reflects the fact that the numerical TDSE cannot handle on-site energies, though the algorithm could still work if the on-site energies are nonzero but sufficiently small compared to the couplings. Conditions 2 and 5 are a prerequisite for the real-valued TDSE (Section S5.1), and thus the dance, which is an implementation of the real-valued TDSE.

In the following sections, we explore the validity of the above conditions. We show that the numerical TDSE can be applied to other Hamiltonians that meet the above conditions: Hamiltonian 1 without some of its NNN couplings (Section S7.2) and the Haldane model (*48*) with inversion symmetry (Section S7.3). In Section S7.4, we explain why the numerical TDSE cannot handle on-site energies (Condition 3), e.g., in the Haldane model (*48*) with broken inversion symmetry. In Section S7.5, we consider an example, namely, Hamiltonian 1 with real-valued next-next-nearest-neighbor (NNNN) couplings, for which the numerical TDSE is not suitable due to violation of Condition 4. Finally, in Section S7.6, we discuss extending the various algorithms reported here to cases where these conditions are not met.

S7.2 Example: applying the numerical TDSE to Hamiltonian 1 without some NNN couplings

As an example, we show that the numerical TDSE (see main text) can be applied to Hamiltonian 1 without some of its NNN couplings (Fig. S9A). Specifically, we consider the removal of the $(m, n) \leftrightarrow (m + 1, n + 1)$ couplings (Eq. 1, third term in big parentheses).

Fig. S9, B to E, shows the exact dynamics for various initial conditions and lattice geometries. The dynamics are qualitatively similar to those of the original Hamiltonian 1 (compare to Fig. 1, B to E, respectively). In particular, the dynamics feature the same key signatures of topological insulators (see main text).

Fig. S10 illustrates the numerical TDSE when applied to the modified Hamiltonian. For the most part, the algorithm qualitatively reproduces the exact dynamics (compare Fig. S10, I to L, and Fig. S9, B to E, respectively). However, a noteworthy discrepancy can be found in the edge dynamics in the presence of site defects: the excitation travels less in the numerical TDSE (Fig. S10J) than in the exact dynamics (Fig. S9C). In the algorithm, the excitation encounters a site defect that leads to two sites $\mathbf{r}_{\text{receiver}}$ (see the two arrows starting at the same site in Fig.

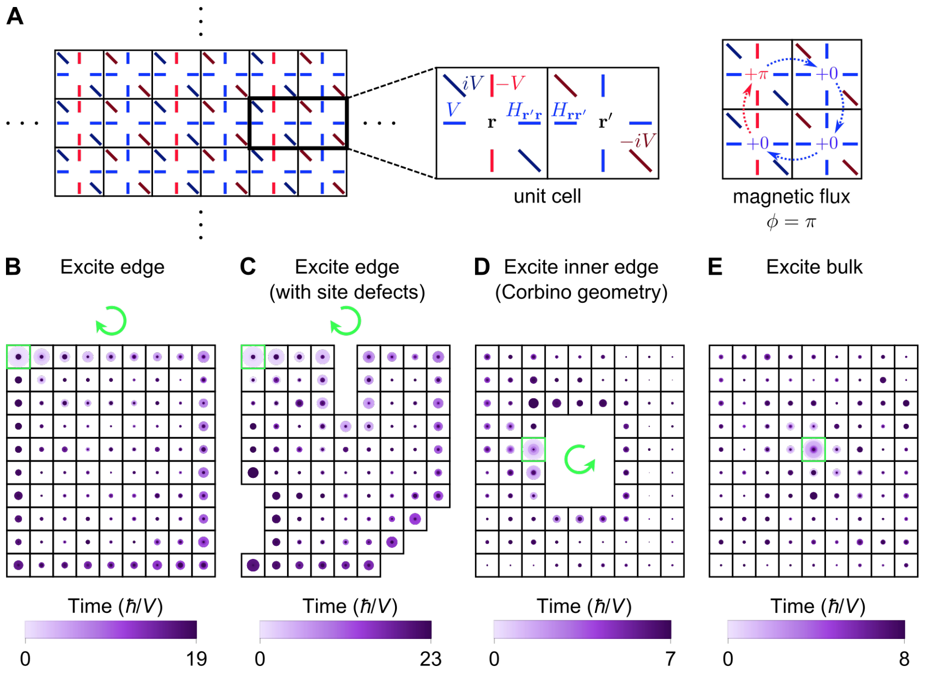

Fig. S9. Dynamics of Hamiltonian 1 without some NNN couplings. Same as Fig. 1 except for Hamiltonian 1 without the $(m, n) \leftrightarrow (m + 1, n + 1)$ couplings (Eq. 1, third term in big parentheses). See Fig. 1 for more details.

S10J), a situation that the numerical TDSE is currently unable to handle (see main text); thus, the algorithm is terminated. Note that this site defect is an arrangement of sites that does not produce multiple $\mathbf{r}_{\text{receiver}}$ when applying the numerical TDSE to the original Hamiltonian 1 (Section S2).

For further insight, we can analyze the site currents, as we have done for the original Hamiltonian 1 (see main text). With some NNN couplings removed, we see that the bulk currents still form a chiral structure (Fig. S10C), from which the edge dynamics can be predicted (Fig. S10, G and H). Thus, the dynamical bulk-boundary correspondence (see main text) holds.

Interestingly, the bulk currents form a loop (Fig. S10C) from which the Chern number naturally emerges. Following Section S4 (in particular, Eq. S8, Fig. S5, and Fig. S6A), we let the horizontal, vertical, and (remaining) NNN hoppings have strength $V_a > 0$, V_b, and V_c', respectively (Fig. S11A). Summing the currents around the loop in counterclockwise fashion (Fig. S11B), and using $V_a > 0$, we find that the sum is proportional to the Chern number $\nu = \text{sgn}(V_b V_c')$ (Eq. S16 and (*39*)).

Since the removal of NNN couplings simply sets site amplitudes to zero, rather than change them from real to imaginary (or vice versa), we can also apply the real-valued TDSE (Section S5.1) to the modified Hamiltonian. Further note that the above results would be qualitatively unchanged if we instead remove the $(m, n) \leftrightarrow (m-1, n+1)$ NNN couplings (Eq. 1, fourth term in big parentheses).

S7.3 Example: applying the numerical TDSE to the Haldane model with inversion symmetry

As another example, we show that the numerical TDSE (see main text) can, with some success, simulate the dynamics of the Haldane model (*48*) where inversion symmetry is obeyed (see Section S7.4 for the case of broken inversion symmetry) but time-reversal symmetry is broken.

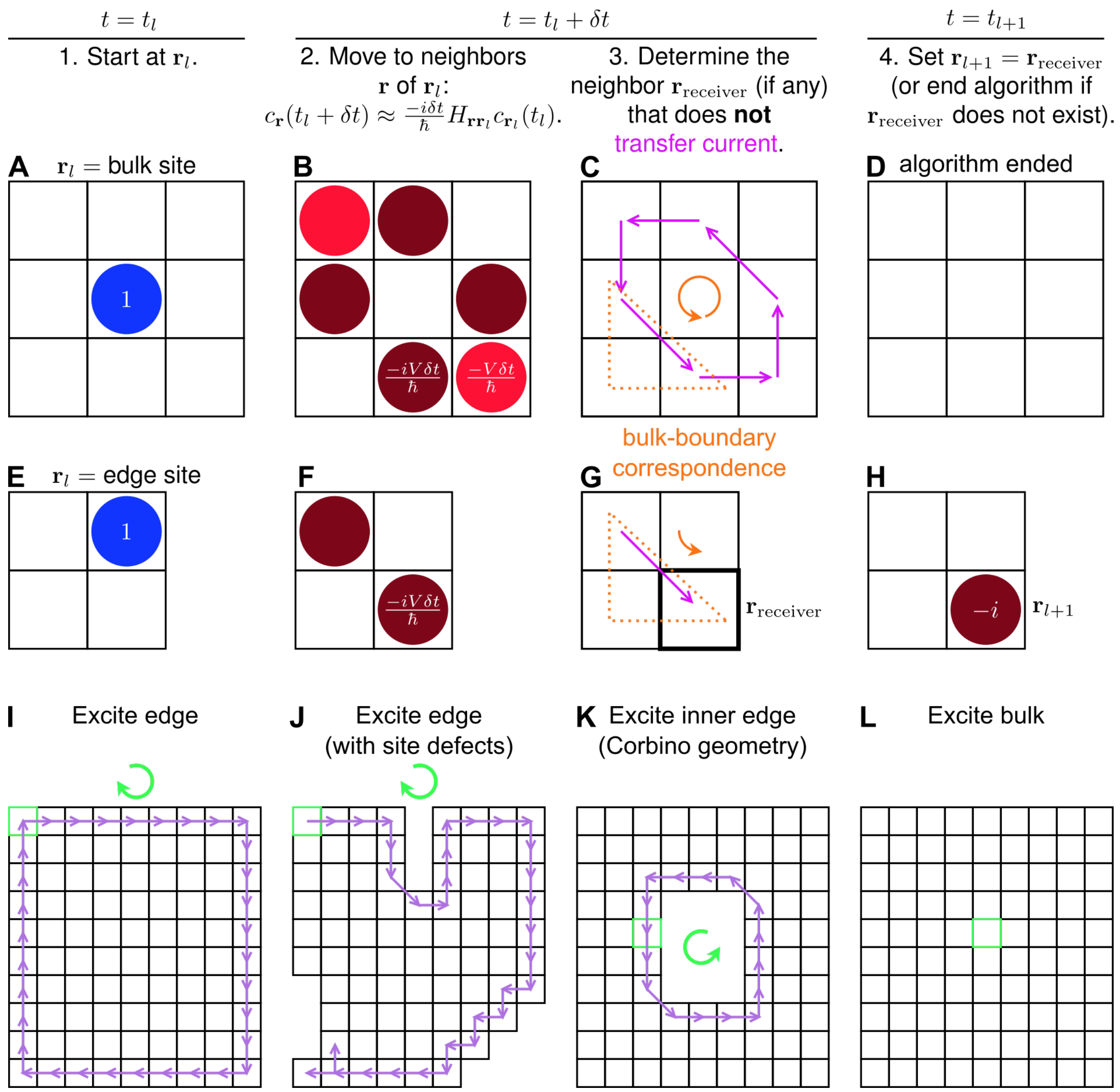

Fig. S10. Numerical TDSE applied to Hamiltonian 1 without some NNN couplings. Same as Fig. 2 except for Hamiltonian 1 without the $(m, n) \leftrightarrow (m+1, n+1)$ couplings (Eq. 1, third term in big parentheses). See Fig. 2 for more details. (J) In contrast to the original Hamiltonian 1 (Fig. 2J), the algorithm is ended before the excitation can make it back to the initial site, since multiple sites are determined to be $\mathbf{r}_{\text{receiver}}$ in the last iteration (see the two arrows starting at the same site).

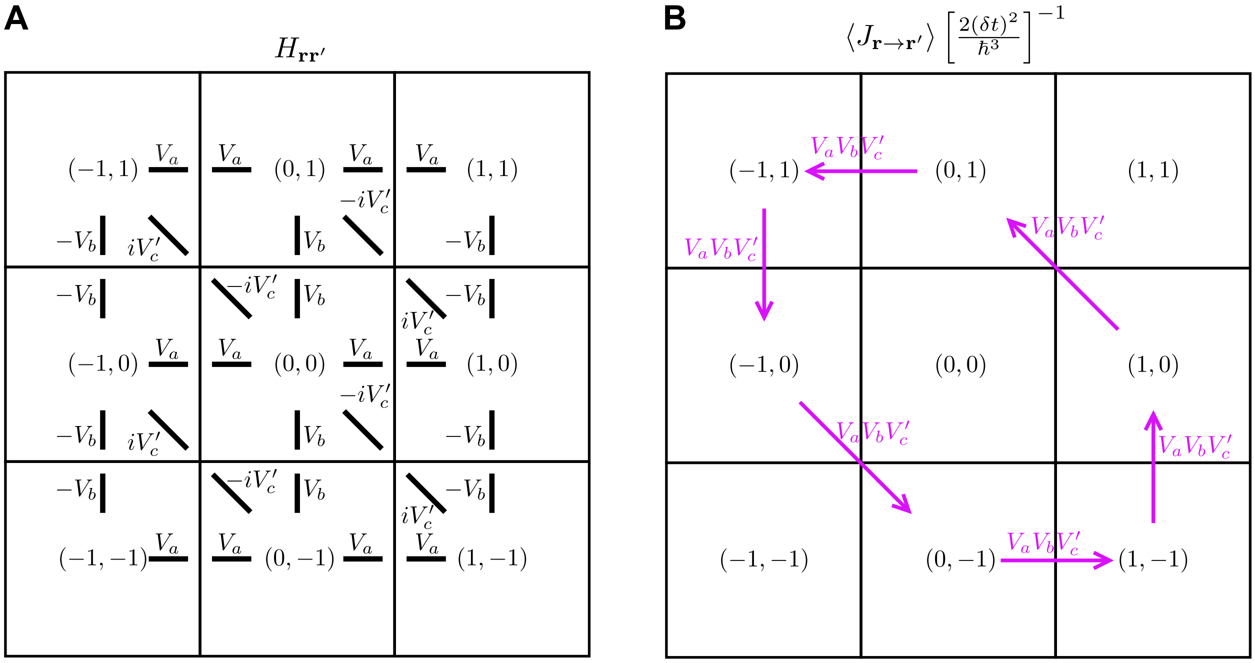

Fig. S11. Couplings and associated currents for the Hamiltonian without some NNN couplings. (A) Couplings ($H_{\mathbf{rr}'}$) for Hamiltonian without the $(m, n) \leftrightarrow (m+1, n+1)$ couplings and (B) associated currents ($\langle J_{\mathbf{r}\to\mathbf{r}'}\rangle \left[\frac{2(\delta t)^2}{\hbar^3}\right]^{-1}$) for the site (0,0) and its neighbors.

Specifically, we consider the Hamiltonian that is pictorially represented in Fig. S12. All hopping amplitudes are taken to have identical magnitude. NNN hopping amplitudes have phase $\pm\pi/2$. Therefore, all hopping amplitudes are either purely real or purely imaginary. Also, it is straightforward to show that, in the numerical TDSE, one sublattice will always have real site amplitudes and the other imaginary. These two properties (see Section S7.1, Conditions 2 and 5) should allow converting the numerical TDSE to the real-valued TDSE (Section S5.1).

Fig. S13 shows the exact dynamics of a localized excitation on a finite lattice (see Materials and Methods). The top and bottom edges have a bearded configuration, while the left and right edges have an armchair configuration. When starting at one end of a bearded edge (Fig. S13A), the excitation primarily propagates down this edge. In contrast, when starting at the other end (Fig. S13B), the excitation spreads considerably down this edge (towards the first end) and the

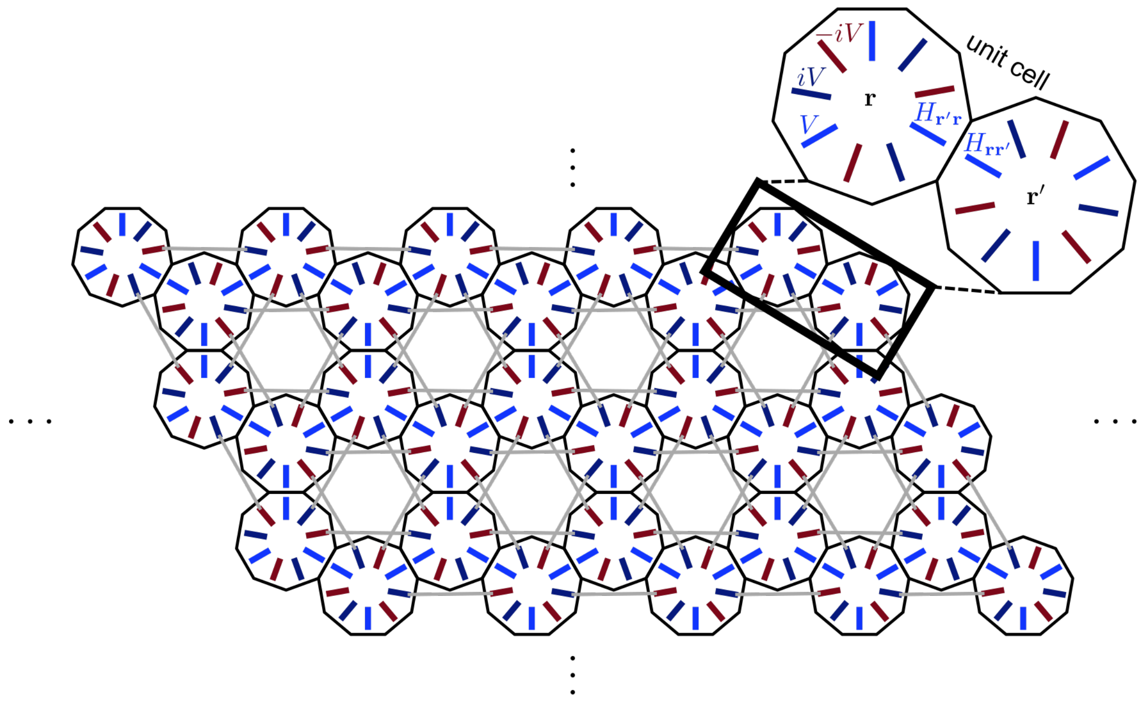

Fig. S12. Hamiltonian of the Haldane model. Pictorial representation of the Hamiltonian (H) of the Haldane model with inversion symmetry obeyed (i.e., without onsite energy) but time-reversal symmetry violated (i.e., with NNN coupling). The gray lines indicate pairs of NNNs. All hopping amplitues have magnitude $V > 0$ ($t_1 = t_2 = V$ in (*48*)). NNN hopping amplitudes have phase $\pm\pi/2$ ($\phi = \pi/2$ in (*48*)).

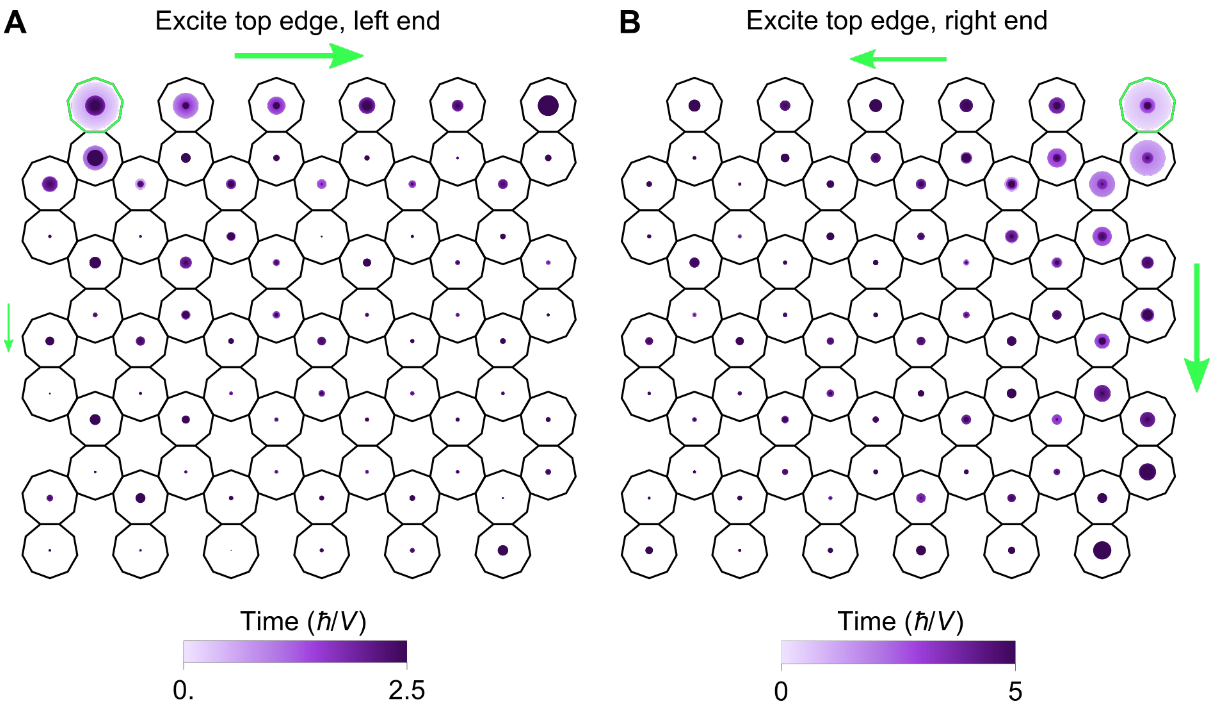

Fig. S13. Edge dynamics of the Haldane model. Dynamics of the Hamiltonian of Fig. S12 on a finite l attice. The top and bottom edges have a bearded configuration, while the left and right edges have an armchair configuration. The system is excited at a site (green box) located at the (A) left and (B) right end of the top edge. Site probabilities at different times are overlaid in chronological order (i.e., later times on top). The probability of the system being at each site is represented by a circle (area $\propto$ probability). The relative extent to which the excitation propagates in selected directions is qualitatively represented by green arrows.

nearest armchair edge, with a slight preference for the latter edge. An excitation in the bulk (Fig. S14) diffuses with little directional selectivity.

For the same lattice geometry and excitation conditions, we now (approximately) simulate the dynamics using the numerical TDSE (see main text and Fig. S15). For an excitation at one end of a bearded edge, the algorithm qualitatively reproduces the edge-localized and largely unidirectional propagation across this edge (compare Figs. S16A and S13A). However, the numerical TDSE does not capture the multidirectional spreading of an excitation on the other end (compare Figs. S16B and S13B). Nevertheless, the slight preference for going down the armchair edge versus the bearded edge (Fig. S13B) seems to appear in the algorithm-generated

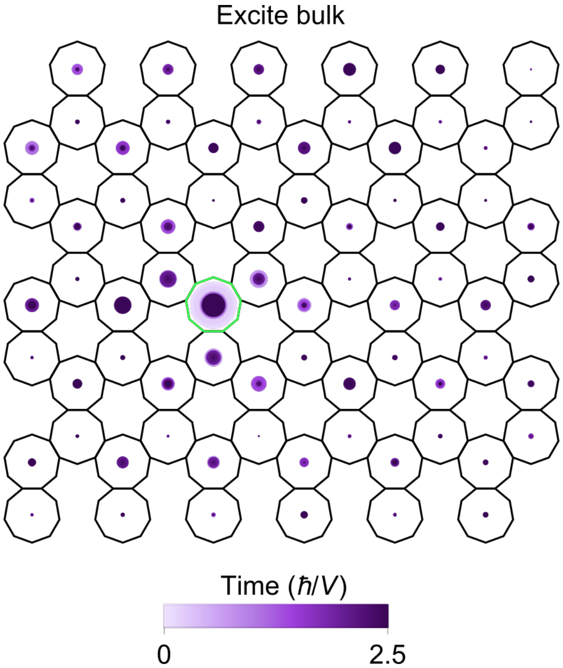

Fig. S14. Bulk dynamics of the Haldane model. Dynamics of the Hamiltonian of Fig. S12 on a finite lattice. The system is excited at a bulk site (green box). See Fig. S13 for more details.

dynamics (Fig. S16B), i.e., as a short-lived trajectory in the preferred direction. Indeed, the absence of bulk propagation as predicted by the numerical TDSE (Fig. S15) is reflective of the low directional selectivity in the exact bulk dynamics (Fig. S14, A to D), as we have also observed for the Harper-Hofstader Hamiltonian of Eq. 1 (see main text). Overall, the numerical TDSE captures some, but not all, qualitative features of the exact dynamics.

As we have pointed out for the Harper-Hofstader Hamiltonian of Eq. 1 (see main text), there exists a dynamical bulk-boundary correspondence when the numerical TDSE is applied to the Haldane model of Fig. S12. This relation can be seen from the current fields generated in Step 3 of the algorithm (see main text and Fig. S16, C, G, and K). Specifically, for an excitation at any bulk site (i.e., surrounded by all possible NNs and NNNs), the current field exhibits a chirality, which is the direction of flow about the excited site from a 3-neighbor cluster to the next (counterclockwise for the Hamiltonian of Fig. S12; see Fig. S15C). For an excitation at

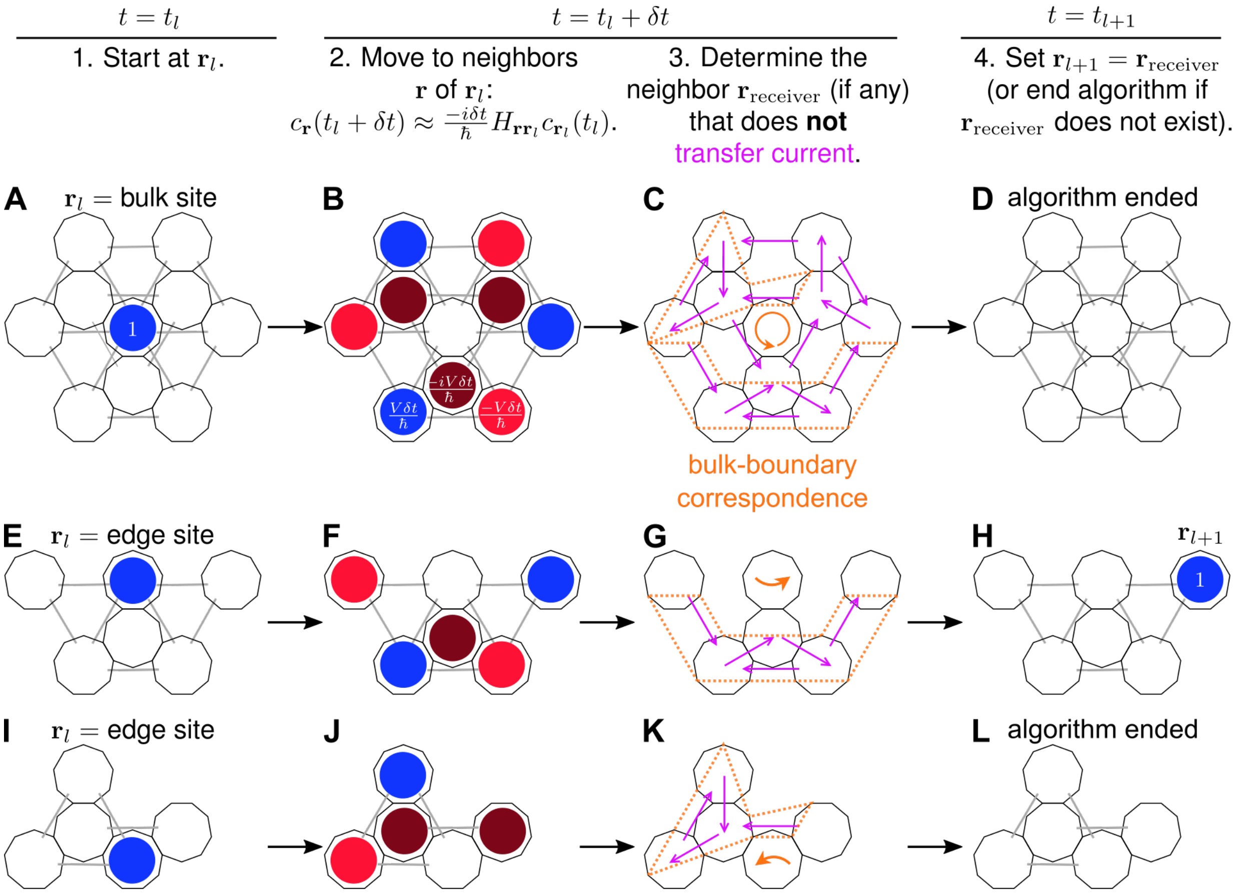

Fig. S15. Numerical TDSE applied to Haldane model. Illustration of the numerical TDSE algorithm when applied to the Haldane model (Fig. S12). The wavefunction starts an iteration at (A to D) a bulk site, (E to H) an edge site where the wavefunction moves to a new site, and (I to L) an edge site where the algorithm ends at the end of the iteration. See Fig. 2, A to H, for more details.

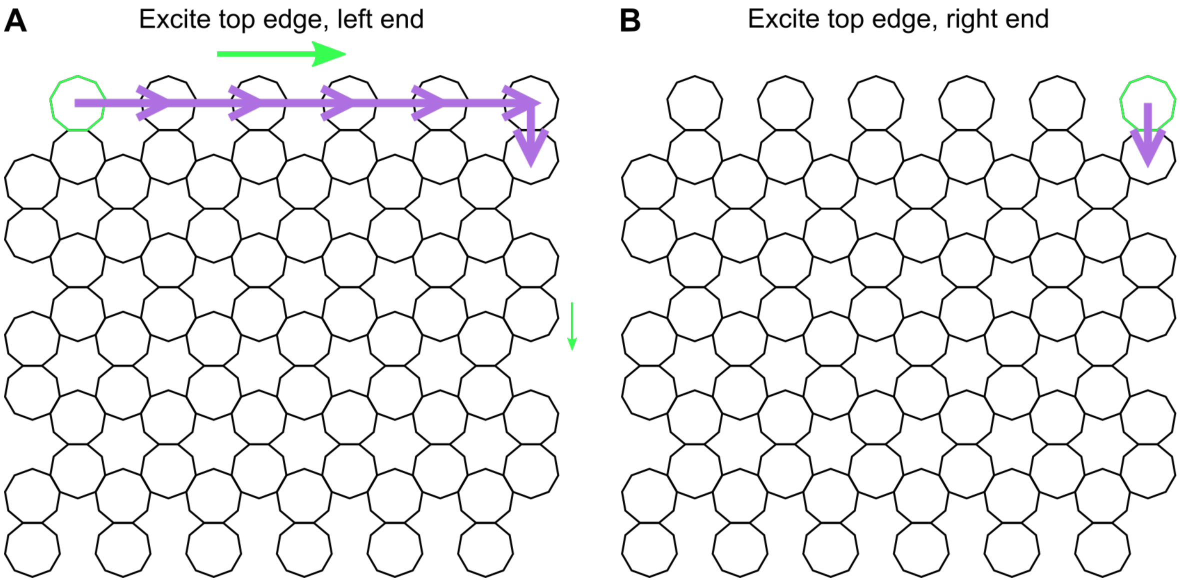

Fig. S16. Discrete-time dynamics of the Haldane model. (**A** and **B**) Dynamics simulated by the numerical TDSE, where the excitation conditions and lattice geometry are those of Fig. S13. For each simulation, the wavefunction starts at the site indicated by the green box. A purple arrow represents the movement of the wavefunction from $\mathbf{r}_l$ (tail) at time step l to $\mathbf{r}_{l+1}$ (head) at time step $l + 1$. The relative extent to which the excitation propagates in selected directions is qualitatively represented by green arrows.

any edge site (i.e., not surrounded by all possible NNs and NNNs), the current field (Fig. S16, G and K) is a subset of the bulk current field (Fig. S16C). Thus, the edge current field and resulting dynamics inherit the chirality of the bulk current field (orange arrows in Fig. S16, C, G, and K).

We also demonstrate a connection between the Chern number and the bulk current field. We do this for a more general Hamiltonian, where the NN hopping amplitudes have magnitude V_1 and the NNN hopping amplitudes have magnitude V_2; the Hamiltonian that we have considered up til now (Fig. S12) corresponds to the special case of $V_1 = V_2 \equiv V$. For the general Hamiltonian, the Chern number is known to be $\nu = \text{sgn}(V_2)$ (*48*). Following Section S4, we consider counterclockwise-oriented loops of neighboring sites, where the loop encloses a bulk site and an integer multiple of the unit cell. Fig. S17 shows representative loops and the corresponding subset of the bulk current field. If we take the sum of the current over loops enclosing two unit cells (Fig. S17, A and B), we see that the sign of the sum is equal to ν, the Chern number. However, if we do the same calculation for a loop enclosing three unit cells (Fig. S17, C), we find that the sum vanishes. Therefore, we have related the bulk current field to the Chern number, though this connection is different from that found for the Harper-Hofstadter Hamiltonian of Eq. S17, where all possible loops enclosing an interger multiple of the unit cell give rise to the Chern number. Given this discrepancy, we would need to explore the correspondence for additional models to understand it intuitively.

S7.4 Why the numerical TDSE cannot handle on-site energies

In this section, we first discuss why the numerical TDSE cannot deal with on-site energies, e.g., in the Haldane model (*48*) with broken inversion symmetry. Specifically, we show that on-site energies have no effect on the current and thus the determination of the site $\mathbf{r}_{\text{receiver}}$ in Step 3 of the algorithm. We then argue that the numerical TDSE can still be used if the on-site

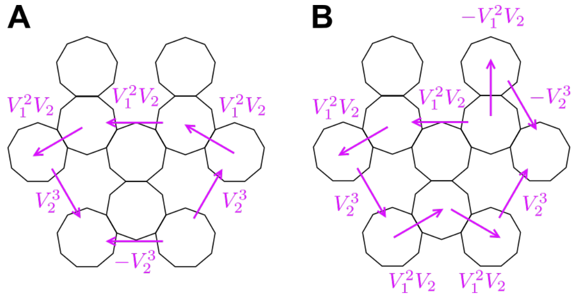

Fig. S17. Loops and associated currents for the Haldane model. Loops (Section S4) for the Haldane model of Fig. S12. The arrows are labeled with their respective currents ($\langle J_{\mathbf{r}\to\mathbf{r}'}\rangle \left[\frac{2(\delta t)^2}{\hbar^3}\right]^{-1}$).

energies are nonzero but sufficiently small compared to the couplings (off-diagonal terms of the Hamiltonian).

If on-site energies are included, then the wavefunction after Step 2 becomes (compare to Eq. 4)

$$|\psi(t_l+\delta t)\rangle = c_{\mathbf{r}_l}(t_l)\left[\left(1-\frac{i\delta t}{\hbar}H_{\mathbf{r}_l\mathbf{r}_l}\right)|\mathbf{r}_l\rangle - \frac{i\delta t}{\hbar}\sum_{\mathbf{r}\in\mathcal{N}(\mathbf{r}_l)} H_{\mathbf{r}\mathbf{r}_l}|\mathbf{r}\rangle\right]. \qquad \text{(S28)}$$

From Eqs. S28 and S1, it is evident that the currents between neighbors of the initial site, $\mathbf{r}_l$, are unaffected by on-site energies, namely, $H_{\mathbf{r}_l\mathbf{r}_l}$. Thus, the site determined to be $\mathbf{r}_{\text{receiver}}$ in Step 3 also remains unchanged. The outcome would be the same even if, for example, Step 3 were modified such that $\mathbf{r}_{\text{receiver}}$ could (in principle) be $\mathbf{r}_l$ itself. Indeed, the current from $\mathbf{r}_l$ to a neighbor $\mathbf{r}$ is

$$\begin{aligned}\langle J_{\mathbf{r}_l\to\mathbf{r}}(t_l+\delta t)\rangle &= \frac{2}{\hbar}\text{Im}\left[\left(-\frac{i\delta t}{\hbar}H_{\mathbf{r}\mathbf{r}_l}\right)^* H_{\mathbf{r}\mathbf{r}_l}\left(1-\frac{i\delta t}{\hbar}H_{\mathbf{r}_l\mathbf{r}_l}\right)\right] \\ &= \frac{2(\delta t)^2}{\hbar^3}|H_{\mathbf{r}\mathbf{r}_l}|^2, \qquad \text{(S29)}\end{aligned}$$

which means that not only does $\mathbf{r}_l$ transfer current to $\mathbf{r}$ (i.e., $\mathbf{r}_{\text{receiver}} \neq \mathbf{r}_l$), but also the magnitude of the current does not depend on $H_{\mathbf{r}_l\mathbf{r}_l}$.

Despite the numerical TDSE not being able to account for the effect of on-site energies, the alogorithm could still work if these terms are not too large. For example, consider the

Haldane model (*48*) with broken inversion symmetry, where this symmetry breaking is induced by nonzero on-site energies. Below a certain magnitude, these terms do not affect the Chern number (± 1) (*48*) and thus the existence of edge states. In this regime, the numerical TDSE can be used as is, at least for the prediction of edge states. However, once the on-site energies become strong enough compared to the coupling (off-diagonal) terms, they must be considered to accurately capture the Chern number going from nonzero to zero, and the current algorithm cannot in its present form account for this topological phase transition.

S7.5 Example: numerical TDSE does not work for Hamiltonian 1 with real NNNN couplings

We consider Hamiltonian 1 with NNNN couplings added. Since the Harper-Hofstadter Hamiltonian is on a square lattice, NNNN couplings correspond to hopping 2 sites horizontally, $(m, n) \to (m \pm 2, n)$, or vertically, $(m, n) \to (m, n \pm 2)$. Specifically, we consider arbitrary real-valued NNNN couplings. Carrying out the numerical TDSE for both bulk and edge excitations, we find that there is no current between each NNNN of the initial site and any other neighbor. Thus, all NNNNs are $\mathbf{r}_{\text{receiver}}$ sites (see main text, numerical TDSE, Step 3), which do not transfer current to any site. For general excitations, this situation corresponds to having multiple $\mathbf{r}_{\text{receiver}}$ sites and therefore cannot be handled by the present form of the numerical TDSE (see main text). In addition, the currents between non-NNNN neighbors of the initial site are the same as for the original Hamiltonian 1, suggesting that the numerical TDSE cannot properly account for real NNNN couplings. These findings result from an "accidental alignment" of the real- or imaginary-valuedness of each type of coupling (NN, NNN, and NNNN).

S7.6 Extending the approach reported here

With some modifications to the algorithms (i.e., numerical and real-valued TDSE), we expect that a dance can be designed for other Hamiltonians, which do not meet the criteria discussed

in Section S7.1.

Consider systems where Condition 1 is not satisfied, i.e., the neighbors of a bulk site are not coupled to each other. Examples include the standard Harper-Hofstadter Hamiltonian (*37, 38*) (i.e., with only nearest-neighbor coupling) and the 1-dimensional Su-Schrieffer-Heeger Hamiltonian (*49*). In these cases, one should modify Step 2 of the numerical TDSE such that the next-nearest neighbors of $\mathbf{r}_l$ are also excited. One possible change is to expand the wavefunction to $O\left((\delta t)^2\right)$ (compare to Eq. 4):

$$|\psi(t_l + \delta t)\rangle \approx \left(1 - \frac{i\delta t}{\hbar}H - \frac{(\delta t)^2}{\hbar^2}H^2\right)|\psi(t_l)\rangle. \tag{S30}$$

Despite the wavefunction being slightly more complicated, the conversion of the numerical TDSE to the real-valued TDSE, and therefore the dance, should remain straightforward.

If Condition 2 is not satisfied, one should still be able to design a dance, so long as the Hamiltonian satisfies the following generalization of Condition 2: the phases of the couplings ($H_{\mathbf{r}\mathbf{r}'}$) make up the ηth roots of unity for some even integer η. For the Hamiltonian studied here (Eq. 1), $\eta = 4$, and the probability amplitudes ($c_\mathbf{r}$) take $2 = 4/2$ possible values after applying f (Eq. 7) to the wavefunction. For general η, one should devise a function, in analogy to f, that maps the probability amplitudes to one of $\eta/2$ values.

Movie S1. Dynamics generated by H for the parameters used in Fig. 1B. The probability of the system being at each site is represented by a circle (area $\propto$ probability).

Movie S2. Dynamics generated by H for the parameters used in Fig. 1C. The probability of the system being at each site is represented by a circle (area $\propto$ probability).

Movie S3. Dynamics generated by H for the parameters used in Fig. 1D. The probability of the system being at each site is represented by a circle (area $\propto$ probability).

Movie S4. Dynamics generated by H for the parameters used in Fig. 1E. The probability of the system being at each site is represented by a circle (area $\propto$ probability).

Movie S5. The dance where the initial dancers are on the lattice edge. Snapshots are shown in Fig. 4B. The chiral edge propagation is indicated by the red arrows moving clockwise over time.

Movie S6. The dance where the initial dancers are on the edge of a lattice with site defects. Snapshots are shown in Fig. 4C. The chiral edge propagation is indicated by the red arrows moving clockwise over time.

Movie S7. The dance where the initial dancer is on the inner edge of a lattice with a hole in the middle (i.e., Corbino geometry). Snapshots are shown in Fig. 4D. The chiral edge propagation is indicated by the red arrow moving counterclockwise over time.

Movie S8. The dance where the initial dancer is in the lattice bulk. Snapshots are shown in Fig. 4E. The lack of bulk propagation is indicated by the red arrow not moving over time.

REFERENCES AND NOTES

1. M. Z. Hasan, C. L. Kane, Colloquium: Topological insulators. *Rev. Mod. Phys.* **82**, 3045–3067 (2010).

2. X.-L. Qi, S.-C. Zhang, Topological insulators and superconductors. *Rev. Mod. Phys.* **83**, 1057–1110 (2011).

3. B. Yan, C. Felser, Topological materials: Weyl semimetals. *Annu. Rev. Condens. Matter Phys.* **8**, 337–354 (2017).

4. K. von Klitzing, G. Dorda, M. Pepper, New method for high-accuracy determination of the fine-structure constant based on quantized hall resistance. *Phys. Rev. Lett.* **45**, 494–497 (1980).

5. D. J. Thouless, M. Kohmoto, M. P. Nightingale, M. den Nijs, Quantized hall conductance in a two-dimensional periodic potential. *Phys. Rev. Lett.* **49**, 405–408 (1982).

6. C.-A. Palma, Topological dynamic matter. *J. Phys. Chem. Lett.* **12**, 454–462 (2020).

7. P. Delplace, J. B. Marston, A. Venaille, Topological origin of equatorial waves. *Science* **358**, 1075–1077 (2017).

8. Z. Wang, Y. Chong, J. D. Joannopoulos, M. Soljačić, Observation of unidirectional backscattering-immune topological electromagnetic states. *Nature* **461**, 772–775 (2009).

9. L. Lu, J. D. Joannopoulos, M. Soljačić, Topological photonics. *Nat. Photonics* **8**, 821–829 (2014).

10. C. He, X. Ni, H. Ge, X.-C. Sun, Y.-B. Chen, M.-H. Lu, X.-P. Liu, Y.-F. Chen, Acoustic topological insulator and robust one-way sound transport. *Nat. Phys.* **12**, 1124–1129 (2016).

11. G. Ma, M. Xiao, C. T. Chan, Topological phases in acoustic and mechanical systems. *Nat. Rev. Phys.* **1**, 281–294 (2019).

12. R. Süsstrunk, S. D. Huber, Observation of phononic helical edge states in a mechanical topological insulator. *Science* **349**, 47–50 (2015).

13. L. M. Nash, D. Kleckner, A. Read, V. Vitelli, A. M. Turner, W. T. M. Irvine, Topological mechanics of gyroscopic metamaterials. *Proc. Natl. Acad. Sci. U.S.A.* **112**, 14495–14500 (2015).

14. T. Kariyado, Y. Hatsugai, Manipulation of dirac cones in mechanical graphene. *Sci. Rep.* **5**, 18107 (2016).

15. K. Schwennicke, J. Yuen-Zhou, Enantioselective topological frequency conversion. *J. Phys. Chem. Lett.* **13**, 2434–2441 (2022).

16. A. F. Ordonez, D. Ayuso, P. Decleva, O. Smirnova, Geometric fields and new enantio-sensitive observables in photoionization of chiral molecules. arXiv:2106.14264 [physics.chem-ph] (2021).

17. N. P. Mitchell, L. M. Nash, D. Hexner, A. M. Turner, W. T. M. Irvine, Amorphous topological insulators constructed from random point sets. *Nat. Phys.* **14**, 380–385 (2018).

18. K. Dasbiswas, K. K. Mandadapu, S. Vaikuntanathan. Topological localization in out-of-equilibrium dissipative systems. *Proc. Natl. Acad. Sci. U.S.A.* **115**, E9031–E9040 (2018).

19. S. Shankar, A. Souslov, M. J. Bowick, M. C. Marchetti, V. Vitelli. Topological active matter. *Nat. Rev. Phys.* **4**, 380–398 (2022).

20. D. Shapira, D. Meidan, D. Cohen, Localization due to topological stochastic disorder in active networks. *Phys. Rev. E* **98**, 012107 (2018).

21. K. Sone, Y. Ashida, T. Sagawa, Exceptional non-hermitian topological edge mode and its application to active matter. *Nat. Commun.* **11**, 5745 (2020).

22. L. Yamauchi, T. Hayata, M. Uwamichi, T. Ozawa, K. Kawaguchi, Chirality-driven edge flow and non-hermitian topology in active nematic cells. arXiv:2008.10852 [cond-mat.soft] (2020).

23. J. Loehr, D. de las Heras, A. Jarosz, M. Urbaniak, F. Stobiecki, A. Tomita, R. Huhnstock, I. Koch, A. Ehresmann, D. Holzinger, T. M. Fischer, Colloidal topological insulators. *Commun. Phys.* **1**, 4 (2018).

24. Q. Yang, H. Zhu, P. Liu, R. Liu, Q. Shi, K. Chen, N. Zheng, F. Ye, M. Yang, Topologically protected transport of cargo in a chiral active fluid aided by odd-viscosity-enhanced depletion interactions. *Phys. Rev. Lett.* **126**, 198001 (2021).

25. H. Feng, J. Wang, Potential and flux decomposition for dynamical systems and non-equilibrium thermodynamics: Curvature, gauge field, and generalized fluctuation-dissipation theorem. *J. Chem. Phys.* **135**, 234511 (2011).

26. A. Murugan, S. Vaikuntanathan, Topologically protected modes in non-equilibrium stochastic systems. *Nat. Commun.* **8**, 13881 (2017).

27. E. Tang, J. Agudo-Canalejo, R. Golestanian, Topology protects chiral edge currents in stochastic systems. *Phys. Rev. X* **11**, 031015 (2021).

28. J. Knebel, P. M. Geiger, E. Frey. Topological phase transition in coupled rock-paper-scissors cycles. *Phys. Rev. Lett.* **125**, 258301 (2020).

29. T. Yoshida, T. Mizoguchi, Y. Hatsugai, Chiral edge modes in evolutionary game theory: A kagome network of rock-paper-scissors cycles. *Phys. Rev. E* **104**, 025003 (2021).

30. T. Yoshida, Y. Hatsugai, Bulk-edge correspondence of classical diffusion phenomena. *Sci. Rep.* **11**, 888 (2021).

31. P. Mehta, J. Rocks, Thermodynamic origins of topological protection in nonequilibrium stochastic systems. arXiv:2206.07761 [cond-mat.stat-mech] (2022).

32. H. Hu, S. Han, Y. Yang, D. Liu, H. Xue, G. G. Liu, Z. Cheng, Q. J. Wang, S. Zhang, B. Zhang, Y. Luo, Observation of topological edge states in thermal diffusion. *Adv. Mater.* **34**, e2202257 (2022).

33. K. Miller, Demonstrating P and S seismic waves (2012); https://youtube.com/watch?v=gjRGIpP-Qfw&ab%005Fchannel=KeithMiller.

34. NOVA PBS Official, How dancing can help you learn science (2016); https://youtube.com/watch?v=d-7AZprW0Rw&ab%005Fchannel=NOVAPBSOfficial.

35. J. L. Silverberg, M. Bierbaum, J. P. Sethna, I. Cohen, Collective motion of humans in mosh and circle pits at heavy metal concerts. *Phys. Rev. Lett.* **110**, 228701 (2013).

36. G. M. Graf, Bulk-edge duality for topological insulators. Presented at Quantum Spectra and Transport, Jerusalem, Israel, 30 June - 4 July, 2013; http://math.huji.ac.il/%7Eavronfest/Graf.pdf.

37. P. G. Harper, Single band motion of conduction electrons in a uniform magnetic field. *Proc. Phys. Soc. A* **68**, 874–878 (1955).

38. D. R. Hofstadter, Energy levels and wave functions of bloch electrons in rational and irrational magnetic fields. *Phys. Rev. B* **14**, 2239–2249 (1976).

39. Y. Hatsugai, M. Kohmoto, Energy spectrum and the quantum hall effect on the square lattice with next-nearest-neighbor hopping. *Phys. Rev. B* **42**, 8282–8294 (1990).

40. O. M. Corbino, Azioni elettromagnetiche doyute agli ioni dei metalli deviati dalla traiettoria normale per effetto di un campo. *Atti R. Accad. Lincei* **1**, 397–420 (1911).

41. B. I. Halperin, Quantized Hall conductance, current-carrying edge states, and the existence of extended states in a two-dimensional disordered potential. *Phys. Rev. B* **25**, 2185–2190 (1982).

42. H. U. Baranger, D. P. DiVincenzo, R. A. Jalabert, A. D. Stone, Classical and quantum ballistic-transport anomalies in microjunctions. *Phys. Rev. B* **44**, 10637–10675 (1991).

43. T. N. Todorov, Tight-binding simulation of current-carrying nanostructures. *J. Phys. Condens. Matter* **14**, 3049–3084 (2002).

44. V. M. Alvarez, J. B. Vargas, L. F. Torres, Non-hermitian robust edge states in one dimension: Anomalous localization and eigenspace condensation at exceptional points. *Phys. Rev. B* **97**, 121401 (2018).

45. Z. Gong, Y. Ashida, K. Kawabata, K. Takasan, S. Higashikawa, M. Ueda, Topological phases of non-hermitian systems. *Phys. Rev. X* **8**, 031079 (2018).

46. J. J. Sakurai, J. Napolitano, *Modern Quantum Mechanics* (Addison-Wesley, 2011).

47. S. Mukamel, *Principles of Nonlinear Optical Spectroscopy* (Oxford Univ. Press, 1995).

48. F. D. M. Haldane, Model for a quantum Hall effect without Landau levels: Condensed-matter realization of the “parity anomaly”. *Phys. Rev. Lett.* **61**, 2015–2018 (1988).

49. W. P. Su, J. R. Schrieffer, A. J. Heeger, Solitons in polyacetylene. *Phys. Rev. Lett.* **42**, 1698–1701 (1979).
